# Systemic and central nervous system metabolic alterations in Alzheimer’s disease

**DOI:** 10.1186/s13195-019-0551-7

**Published:** 2019-11-28

**Authors:** Vera van der Velpen, Tony Teav, Héctor Gallart-Ayala, Florence Mehl, Ioana Konz, Christopher Clark, Aikaterini Oikonomidi, Gwendoline Peyratout, Hugues Henry, Mauro Delorenzi, Julijana Ivanisevic, Julius Popp

**Affiliations:** 1 Metabolomics Unit, Faculty of Biology and Medicine, University of Lausanne, Lausanne, Switzerland; 2 Old Age Psychiatry, Department of Psychiatry, Lausanne University Hospital, Lausane, Switzerland; 3 Clinical Chemistry Laboratory, Department of Biomedicine, Lausanne University Hospital, Lausane, Switzerland; 4 Translational Bioinformatics and Statistics, Department of Oncology, Swiss Cancer Center Leman (SCCL), University of Lausanne, Lausanne, Switzerland; 5 Bioinformatics Core Facility, SIB Swiss Institute of Bioinformatics, Lausanne, Switzerland; 6 Department of Psychiatry, Lausanne University Hospital, Route du Mont, 1008 Prilly-Lausanne, Switzerland

**Keywords:** Alzheimer’s disease, Metabolomics, Energy metabolism, Tryptophan pathway, CSF AD biomarkers

## Abstract

**Background:**

Metabolic alterations, related to cerebral glucose metabolism, brain insulin resistance, and age-induced mitochondrial dysfunction, play an important role in Alzheimer’s disease (AD) on both the systemic and central nervous system level. To study the extent and significance of these alterations in AD, quantitative metabolomics was applied to plasma and cerebrospinal fluid (CSF) from clinically well-characterized AD patients and cognitively healthy control subjects. The observed metabolic alterations were associated with core pathological processes of AD to investigate their relation with amyloid pathology and tau-related neurodegeneration.

**Methods:**

In a case-control study of clinical and biomarker-confirmed AD patients (*n* = 40) and cognitively healthy controls without cerebral AD pathology (*n* = 34) with paired plasma and CSF samples, we performed metabolic profiling, i.e., untargeted metabolomics and targeted quantification. Targeted quantification focused on identified deregulated pathways highlighted in the untargeted assay, i.e. the TCA cycle, and its anaplerotic pathways, as well as the neuroactive tryptophan and kynurenine pathway.

**Results:**

Concentrations of several TCA cycle and beta-oxidation intermediates were higher in plasma of AD patients, whilst amino acid concentrations were significantly lower. Similar alterations in these energy metabolism intermediates were observed in CSF, together with higher concentrations of creatinine, which were strongly correlated with blood-brain barrier permeability. Alterations of several amino acids were associated with CSF Amyloidβ1–42. The tryptophan catabolites, kynurenic acid and quinolinic acid, showed significantly higher concentrations in CSF of AD patients, which, together with other tryptophan pathway intermediates, were correlated with either CSF Amyloidβ1–42, or tau and phosphorylated Tau-181.

**Conclusions:**

This study revealed AD-associated systemic dysregulation of nutrient sensing and oxidation and CNS-specific alterations in the neuroactive tryptophan pathway and (phospho)creatine degradation. The specific association of amino acids and tryptophan catabolites with AD CSF biomarkers suggests a close relationship with core AD pathology.

Our findings warrant validation in independent, larger cohort studies as well as further investigation of factors such as gender and APOE genotype, as well as of other groups, such as preclinical AD, to identify metabolic alterations as potential intervention targets.

## Introduction

In Alzheimer’s disease (AD), glucose hypometabolism is considered a typical feature of the disease at clinical stages, indicating the loss of neuronal function in specific brain regions [1]. Cerebral glucose hypometabolism, characterized by impaired glucose uptake and utilization related to brain insulin resistance [2, 3], and progressive mitochondrial dysfunction with aging [4] have both been recently associated with AD and suggest involvement of energy metabolism alterations in AD pathophysiology. Importantly, these alterations in early AD may occur both at the central nervous system (CNS) and the systemic level and play a role in clinical disease progression [5, 6]. Despite these observations, the extent and significance of CNS and systemic metabolic alterations in AD remain poorly understood. Therefore, further and in-depth characterization of metabolic alterations to unravel potential new targets for therapeutic intervention is needed. Metabolomics is a powerful phenotyping technology, which allows to systematically identify and quantify the active small molecule-metabolite complement of cells, tissues, or biofluids and provide a sensitive and highly specific multiparametric measure of disease phenotype at the molecular level [7–14].

A few recent metabolomics data-driven studies in subjects with clinically defined AD support the view of AD as an energy metabolism and metabolic signaling disorder by describing metabolic alterations in amino acid, acylcarnitine, sphingolipid, and lipid metabolism [6, 15–19]. While many alterations were observed, the results of these studies are inconsistent regarding the amplitude and direction of the alterations and limited by the lack of quantitative data and shortcomings in study design. Importantly, the link between systemic and CNS changes and their relationships with the AD core brain pathology, i.e., amyloid accumulation and tau pathology, remain largely unexplored.

Here, we present a case-control study using paired plasma and cerebrospinal fluid (CSF) samples from AD patients and cognitively healthy controls characterized using combined clinical and biomarker-based criteria [20] to which we applied state-of-the-art metabolomic approaches. We used a thorough stepwise approach from untargeted profiling and pathway analysis to targeted absolute quantification to determine the presence and magnitude of metabolic alterations and metabolite concentration ranges. By linking systemic and CNS metabolic alterations and by addressing relationships between metabolites and markers of core AD pathology, i.e., amyloid accumulation and tau-related neurodegeneration, this study contributes to the functional understanding of AD pathophysiology.

## Methods

### Subjects

Participants with AD were recruited among patients with cognitive impairment attending the Memory Clinics of the Department of Psychiatry and the Department of Clinical Neurosciences at Lausanne University Hospital for the diagnosis of their complaints [21]. Control subjects were recruited by announcements and word of mouth. All participants underwent a comprehensive medical, neuropsychological, and psychosocial evaluation, as well as brain MRI or CT scans, and venous and lumbar punctures. The MRI and CT scans were used to exclude cerebral pathologies possibly interfering with the cognitive performance.

The AD group (*n* = 40) consisted of subjects with both cognitive impairment established with a Clinical Dementia Rating (CDR) of 0.5 or 1 *and* an AD CSF biomarker profile (see Additional file 2: methods, section 1.3). The control group (*n* = 34) consisted of subjects without cognitive impairment (CDR = 0) and with normal CSF biomarker profile (Table 1). Subjects with cognitive impairment and a non-AD CSF biomarker profile or with normal cognition and an AD CSF profile were not included.

**Table 1:** Clinical characteristics of the cohort

Clinical characteristics	AD (*n* = 40)	Control (*n* = 34)	*P* value^a^
Female, *n* (%)	24 (60.00)	23 (67.65)	0.6098
BMI, kg/m^2^, mean ± SD	23.83 ± 3.06	24.60 ± 4.01	0.3637
Age, year, mean ± SD	74.88 ± 6.38	65.35 ± 6.17	< 0.0001
Cognitive function			
MMSE, mean ± SD	24.40 ± 4.15	28.71 ± 1.29	< 0.0001
CDR, mean ± SD	0.65 ± 0.30	0.00 ± 0.00	< 0.0001
AD CSF biomarkers			
Aβ1–42, pg/ml, mean ± SD	556.22 ± 115.39	979.12 ± 164.38	< 0.0001
Tau, pg/ml, mean ± SD	715.70 ± 300.05	196.18 ± 59.72	< 0.0001
pTau-181, pg/ml, mean ± SD	91.95 ± 23.84	42.91 ± 10.51	< 0.0001
Biochemical measures			
ApoEε4, *n* (%ε4)	26 (65.00)	6 (17.64)	< 0.0001
Qalb, mean ± SD	6.69 ± 3.76	5.27 ± 1.82	0.0474

^a^*P* value represents result of *t*-test comparing AD and control group for continuous variables and chi-square test for categorical variables (male/female frequency and ApoEε4 distribution). *MMSE* Mini-Mental State Exam, *CDR* Clinical Dementia Rate, *Qalb* quotient albumin or plasma/CSF albumin ratio

### AD diagnosis and cognitive assessments

The diagnosis of MCI or mild dementia of AD type was based on neuropsychological and clinical evaluation made by a consensus conference of neuropsychologists, psychiatrists, and/or neurologists prior to inclusion into the study, as described elsewhere [22] and detailed in Additional file 2: methods (section 1.1) together with the performed cognitive assessments.

### Sample collection, APOE genotyping, and CSF AD biomarker assessment

CSF and plasma samples were obtained as previously described [22], and subsequently, CSF AD biomarkers Aβ1–42, tau, and pTau-181 were measured using ELISA (Fujirebio, Ghent, Belgium). The APOE genotype was determined as previously described [21]. Brief details of both procedures are outlined in the Additional file 2: methods (section 1.2 and 1.3).

### State-of-the-art untargeted and targeted metabolic profiling

Materials and detailed methods are outlined in Additional file 2: methods (section 1.4).

### Untargeted profiling

Following the extraction with MeOH:ACN, plasma and CSF sample extracts were subjected to LC-MS analysis using the 6550 iFunnel Q-TOF MS interfaced with 1290 UHPLC (Agilent Technologies, Basel, CH) as previously described [23]. The data were processed using XCMS Online [24] and signal drift correction was applied and metabolite features showing analytical variability > 30% were removed. Putative identification was done in XCMS Online linked to METLIN metabolite database [25], and metabolite identities were further validated with tandem MS experiments as previously described [23, 26].

### Broad-scale targeted profiling

In parallel with untargeted profiling, broad-scale targeted screening was performed with a focus on intermediates involved in multiple central carbon pathways (242 metabolites) using a 6495 iFunnel triple quadrupole system (QqQ, Agilent Technologies, Basel, CH) interfaced with the 1290 UHPLC system. Data was acquired in dynamic multiple reaction monitoring mode (dMRM, cycle time 600 ms). Data processing was done using MassHunter Quantitative Analysis (for QqQ, version B.07.01/ Build 7.1.524.0, Agilent Technologies). Signal drift correction was applied on the QC samples [27], and metabolites with CV > 20% were discarded.

### Pathway analyses

Pathway analyses were performed using MetaboAnalyst 3.0 [28], and the human pathways from the *Homo sapiens* Kyoto Encyclopedia of Genes and Genomes (KEGG) database were used as the source of pathway topologies to deduce pathways of interest for the absolute quantification method. Pathway impact has been calculated as the sum of the importance measures (i.e., centrality measure within a given metabolic network) of the matched metabolites normalized by the sum of the importance measures of all metabolites in each pathway [29] (for further details, see Additional file 2: methods, section 1.4.5).

### Targeted quantification of tricarboxylic acid (TCA) cycle intermediates, tryptophan breakdown products, and other amino acids and acylcarnitines

Absolute quantification was performed using the 6495 QqQ mass spectrometer interfaced with the 1290 UHPLC, operated in the dMRM mode. In brief, aliquots of calibrators, plasma, or CSF were extracted by the addition of internal standard mixtures (in MeOH) after which the sample was directly injected for LC-MS/MS analysis (transitions are provided in Additional file 1: Table S1). Stable isotope-labeled analogues were used as internal standards to determine the response factor while correcting for extraction yield and matrix effect. Data processing was done using MassHunter Quantitative Analysis.

### Statistical analysis

Group comparison was performed with the absolute concentration data, which was done using a parametric *t*-test with a *p* value significance cut-off 0.05 (FDR < 0.25). Additional testing was performed to assess (1) gender differences, (2) the CSF/plasma ratio of metabolite concentrations, (3) the influence of blood-brain barrier (BBB) permeability, and (4) the potential confounding effect of age and ApoE4, for which *p* < 0.05 was considered significant. Associations between metabolite concentrations and single CSF AD biomarker concentrations (t-tau, pTau-181, and Aβ1–42) were evaluated using simple and multiple linear regression analysis for the AD group only. Details of these analyses are outlined in Additional file 2: methods (section 1.5).

## Results

### Clinical characteristics of controls and subjects with Alzheimer’s disease

For this study, *n* = 40 well-characterized AD patients and *n* = 34 cognitively healthy controls were selected (Table 1). As about 20% of the patients with a clinical criteria-based diagnosis of AD have no cerebral AD pathology [30], we only included AD subjects with both clinical signs of (prodromal) AD and a CSF AD biomarker profile (pTau-181/Aβ1–42 ratio > 0.078) [20]. Conversely, a significant percentage of elderly persons without any clinical signs of AD have cerebral AD pathology [30]. In this study, we only included control subjects who were cognitively healthy and had normal CSF AD biomarker profiles. The AD patient and control groups did not differ in male/female distribution and BMI, but the AD patients were generally older. In addition, the two populations had a different frequency of the ApoEε4 allele, a known risk factor for AD, and a marginally significant different CSF/serum albumin ratio (Qalb), considered here as a marker of blood-brain barrier permeability [31].

### Metabolic profiling highlights disrupted core energy metabolism and tryptophan pathway alterations in Alzheimer’s disease

**Fig. 1:**
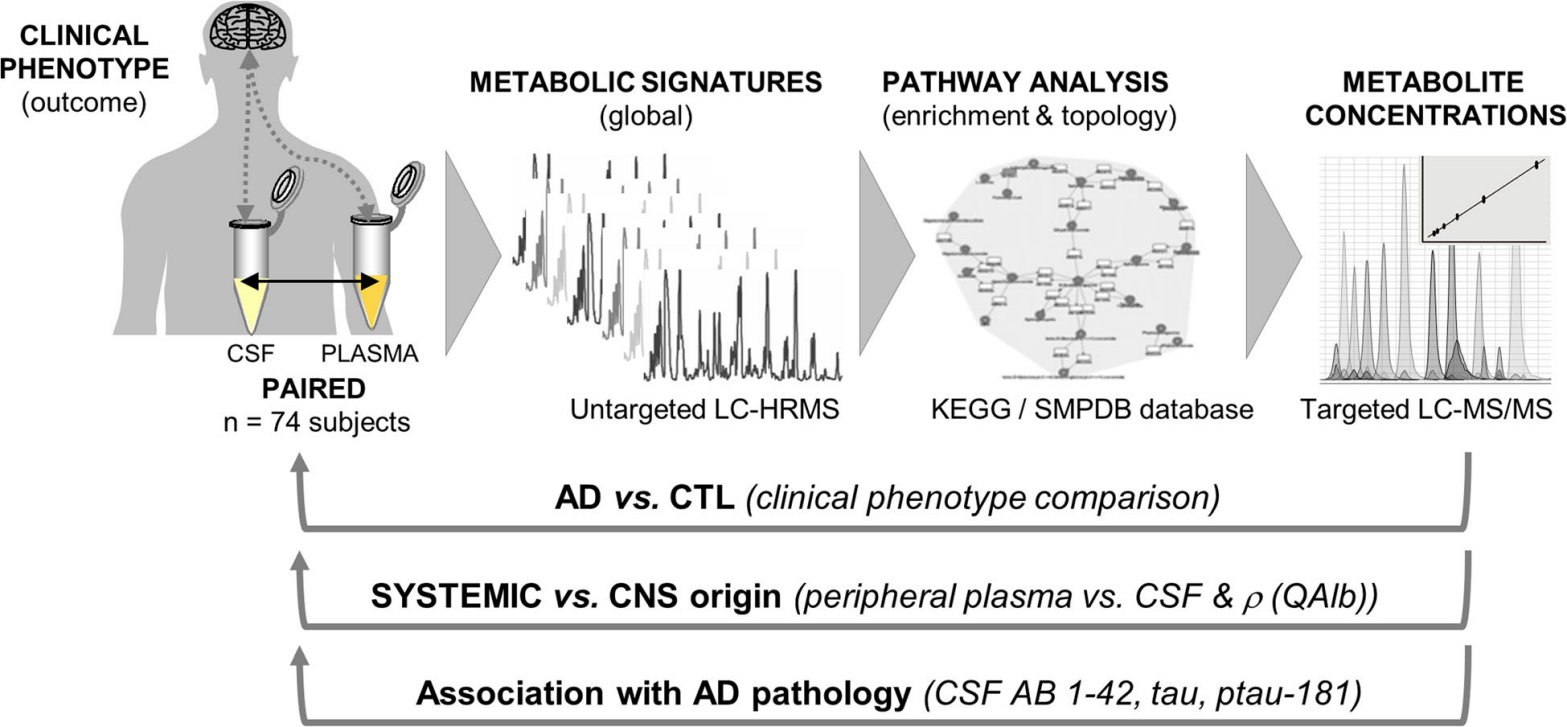
Study design and metabolic profiling workflow. Plasma and CSF samples were collected concomitantly, from the same subject. Metabolic signatures acquired by the untargeted profiling were explored using the pathway enrichment and topology analysis to identify the biochemical pathways affected in AD. Targeted quantification of metabolites implicated in these identified affected pathways was then performed to obtain the accurate and precise measurement of metabolite concentrations. The clinical phenotype comparison was followed by paired blood plasma vs. CSF comparison and correlation with QAlb to assign the origin of the observed changes. Finally, the associations with known CSF markers of AD pathology were investigated to link the identified changes at the metabolite and pathway level with the clinical outcome. LC-HRMS – liquid chromatography coupled to high-resolution mass spectrometry, LC-MS/MS – liquid chromatography coupled to tandem mass spectrometry, KEGG – Kyoto Encyclopedia of Genes and Genomes, SMPDB – Small Molecule Pathway Data Base

State-of-the-art metabolic profiling, including untargeted profiling and quantitative targeted analysis, was applied to identify changes at the metabolite and pathway level in AD, to quantify their amplitude and to determine their origin (systemic vs. CNS) and association with distinct AD pathological processes (Fig. 1).

Untargeted profiling pointed towards significant alterations in the amino acid metabolism and energy-producing fatty acid oxidation (i.e., acylcarnitine levels) in plasma and in CSF of AD patients (Additional file 1: Table S1). These differences were confirmed by further broad-scale targeted screening that allowed us to reveal several additional changes in the levels of glycolysis and tryptophan and kynurenine pathway intermediates (Additional file 1: Table S2). Pathway over-representation combined with topology analysis, which considers the position and biological relevance of profiled metabolites within their respective pathways, showed significantly enriched tryptophan and histidine metabolism as well as beta-oxidation pathway in plasma. In CSF, enriched tryptophan and lysine metabolism were highlighted, as well as glycolysis/gluconeogenesis, the pentose phosphate pathway, and carnitine synthesis (*P* < 0.05, Additional file 1: Table S3). Following these results, we quantified in an absolute fashion different intermediates in the TCA cycle as a hub of energy metabolism, and its anaplerotic pathways, i.e., fatty acid oxidation and specific amino acid pathways. The downstream products of tryptophan metabolism were also quantified due to high enrichment and impact score of tryptophan metabolism in both plasma and CSF in the pathway analysis (*P* < 0.002, Impact > 0.22).

**Fig. 2:**
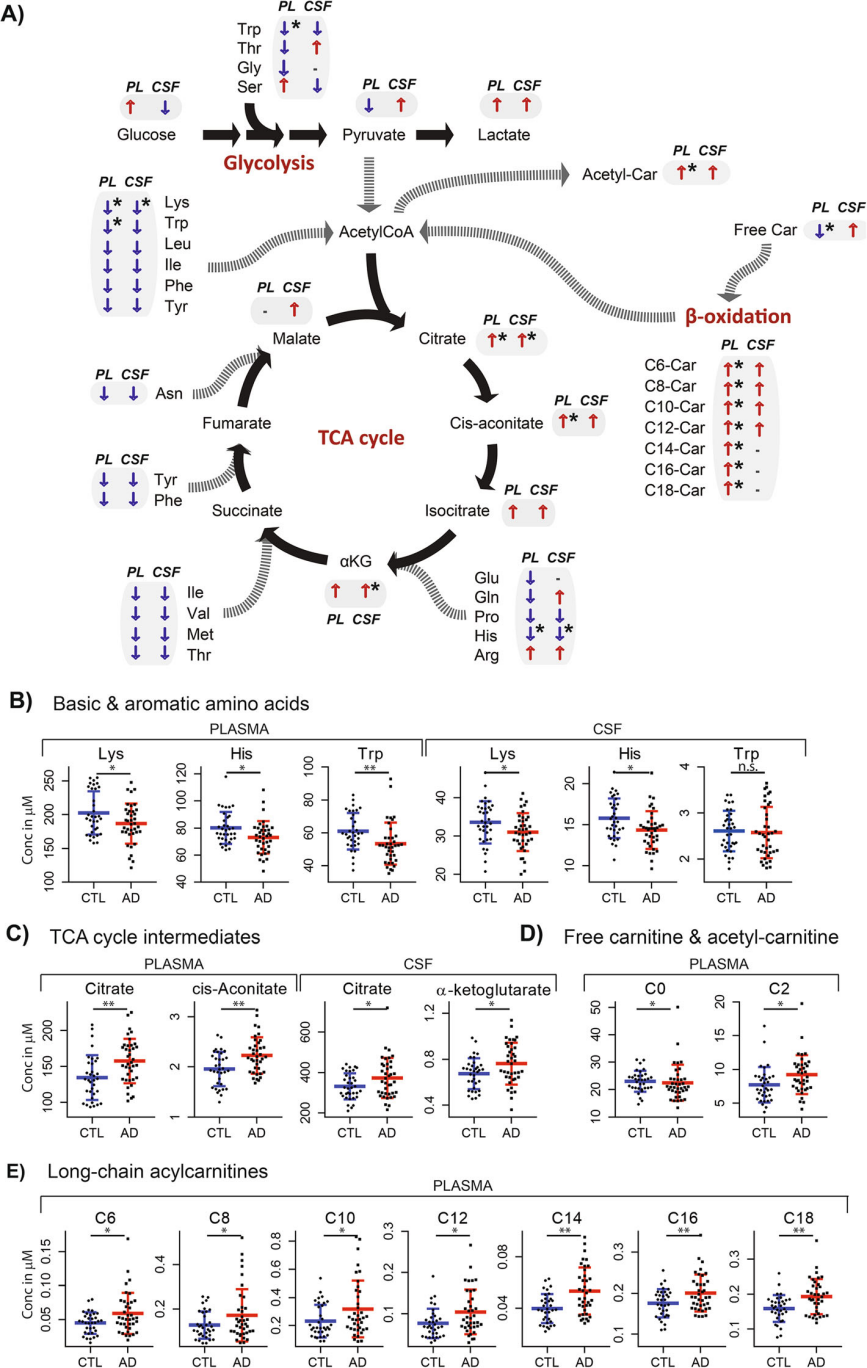
Systemic and central nervous system alterations in AD in the energy metabolism hub; the TCA cycle and its anaplerotic pathways (i.e., amino acid metabolism, glycolysis and beta-oxidation). For **a** direction of metabolite alterations in AD patients versus control in plasma (PL) and CSF, ↑ higher concentrations in AD vs control, ↓ lower concentrations in AD vs control, “-“ indicates “not detected” or below limit of quantification, * statistically significant higher or lower concentrations in AD vs control *P* < 0.05 (T-test). For **b** to **e**, * statistically significant *P* < 0.05 (*T*-test), **P < 0.01, n.s. not significant

Following absolute quantification, intermediates from the TCA cycle had higher concentrations in AD patients compared to control subjects in both plasma and CSF. Significantly higher concentrations of citrate were observed in AD, in both plasma (%diff_plasma_ = 17.2%, *P* = 0.002) and in CSF (%diff_CSF_ = 12.5%, *P* = 0.036). In addition, cis-aconitate (%diff = 14.0%, *P* = 0.002) and α-ketoglutarate (%diff = 13.0%, *P* = 0.020) were significantly increased in AD in plasma and in CSF, respectively (Fig. 2, Additional file 1: Table S5). The glucogenic and ketogenic amino acids, producing intermediates that feed into the TCA cycle, had lower concentrations in AD patients in both plasma and CSF. Significantly lower concentrations were observed for the basic amino acids, lysine (%diff_plasma_ = − 8.6%, *P*_plasma_ = − 0.032; %diff_CSF_ = − 8.3%, *P*_CSF_ = 0.040) and histidine (%diff_plasma_ = − 9.7%, *P*_plasma_ = 0.014; %diff_CSF_ = − 10.1%, *P*_CSF_ = 0.010), as well as tryptophan in plasma (%diff = − 14.2%, *P* = 0.009). Oppositely, significantly higher concentrations of creatinine were observed in CSF of AD patients (%diff = 15.4%, *P* = 0.00001).

Acylcarnitines, the transporter variants of fatty acid oxidation intermediates that fuel the TCA cycle by generating AcetylCoA via beta-oxidation, showed significantly higher concentrations in plasma of AD patients compared to control subjects (Fig. 2, Additional file 1: Table S5). These were medium- and long-chain acylcarnitines with an acyl-chain of C6 (%diff = 31.4%, *P* = 0.016), C8 (%diff = 34.8%, *P* = 0.048), C10 (%diff = 37.0%, *P* = 0.029), C12 (%diff = 36.4%, *P* = 0.012), C14 (%diff = 34.4%, *P* = 0.0003), C16 (%diff = 14.2%, *P* = 0.009), and C18 (%diff = 21.1%, *P* = 0.002). In addition, the concentration of acetylcarnitine (C2) was significantly higher (%diff = 19.2%, *P* = 0.025), whilst the free pool of carnitine (C0) in plasma was significantly lower (%diff = − 12.4%, *P* = 0.026) in AD patients. In CSF, the same trend of accumulation in AD was observed for acylcarnitines with a chain length between C6 and C12, whereas the long-chain acylcarnitines were below the limit of quantification.

It is worth noting that for the majority of measured metabolites, the observed differences were more pronounced in women than in men as illustrated in Additional file 1: Figure S1.

**Fig. 3:**
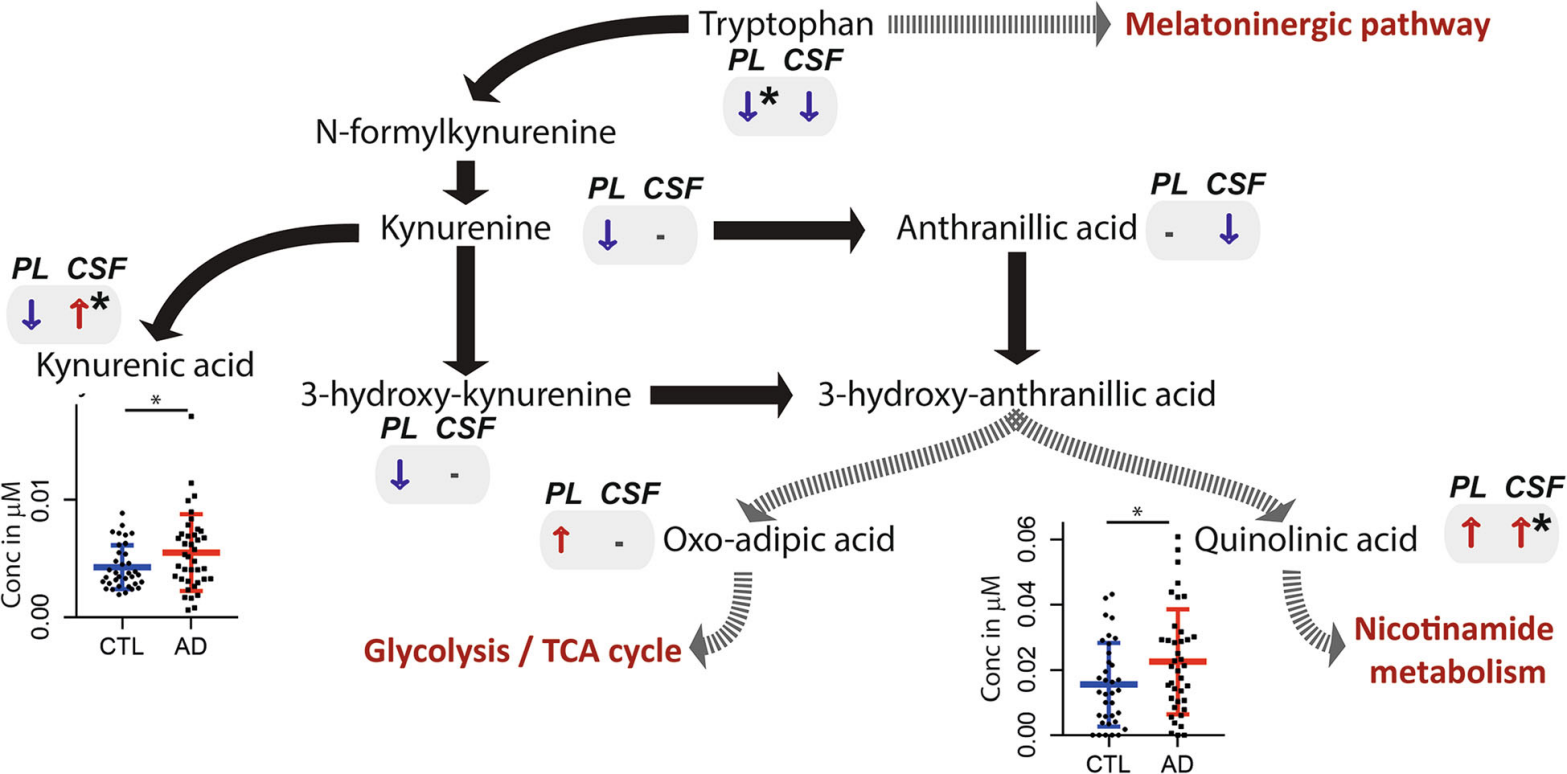
Systemic and central nervous system alterations in products of tryptophan breakdown in AD. Direction of metabolite alterations in AD patients versus control in plasma (PL) and CSF; ↑ higher concentrations in AD vs control, ↓ lower concentrations in AD vs control, “-“ indicates “not detected” or below limit of quantification, * statistically significant higher or lower concentrations in AD vs control *P* < 0.05 (T-test)

Tryptophan pathway intermediates, including tryptophan itself (%diff = − 14.2%, *P* = 0.009), had generally lower concentrations in plasma of AD patients. In CSF, while tryptophan concentrations were lower, the downstream products of tryptophan degradation, i.e., kynurenic acid (%diff = 29.1%, *P* = 0.046) and quinolinic acid (%diff = 45.5%, *P* = 0.040) were significantly higher in AD patients compared to control subjects (Fig. 3), a difference driven by females only (*P*_kynurenic acid_ = 0.0035, *P*_quinolinic acid_ = 0.0069, Additional file 1: Figure S1).

### Correlations of altered metabolites in CSF with BBB permeability

**Fig. 4:**
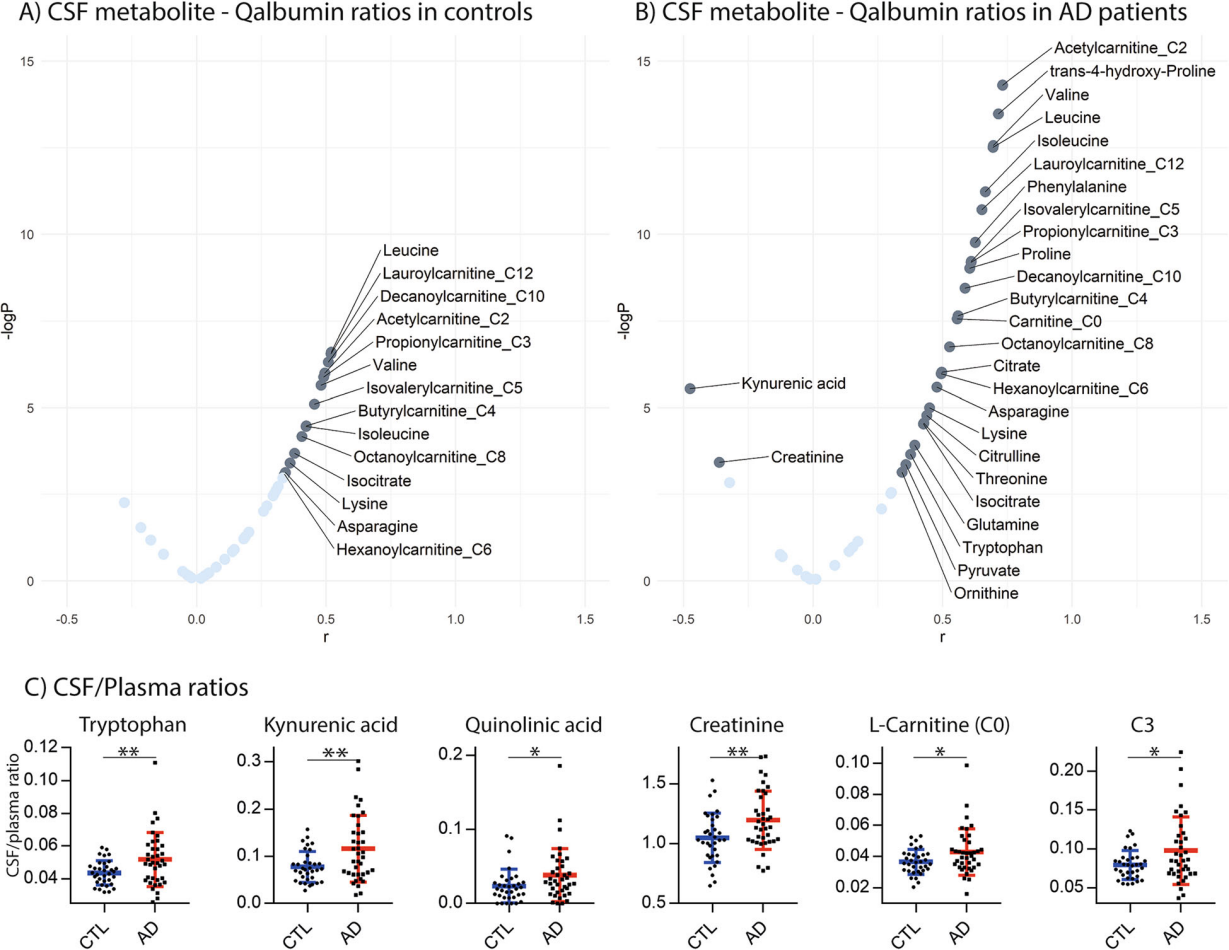
Correlations of metabolite concentrations in CSF with Qalb in control (**a**) and AD patients (**b**) and boxplots of metabolites with significantly different CSF/plasma ratios between control and AD patients (**c**). For **a** and **b**, significantly different metabolites in dark blue with –log*P* value > 3 (represents *P* value < 0.05). For **c**, *P < 0.05 and **P < 0.001

Using the Qalb as a measure of blood-brain barrier integrity, we found that amino acid and acylcarnitine concentrations in CSF showed a significant positive correlation with Qalb in control subjects. This positive correlation was even more pronounced and significant in AD patients (for *P* < 0.001, *r* > 0.6, Fig. 4a, b, Additional file 1: Table S6). While majority of amino acids and acylcarnitines showed positive correlation with Qalb, kynurenic acid and creatinine were negatively correlated with Qalb. Furthermore, both these metabolites, as well as tryptophan, quinolinic acid, and two (acyl)carnitines (C0 and C3) had significantly higher CSF/plasma ratios in AD patients compared to control subjects (Fig. 4c).

### Metabolite alterations associated with CSF biomarkers of core AD pathology

**Fig. 5:**
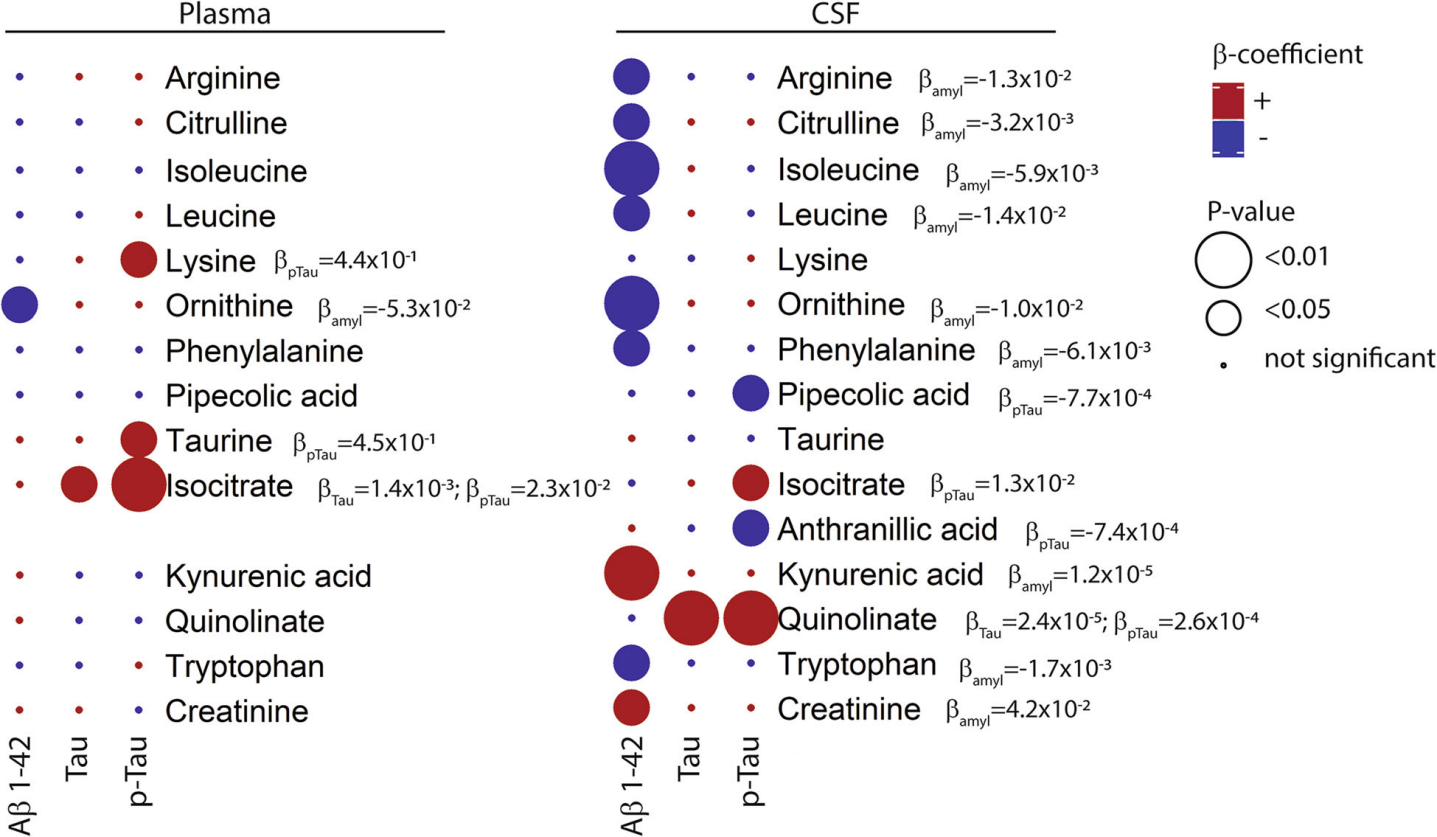
Associations of plasma (left) and CSF (right) metabolite concentrations with core AD pathology as measured by CSF biomarker concentrations. Results from linear regression analysis are presented; colors represent beta-coefficients of the CSF biomarker estimate (red for positive association, blue for negative association), circle size represents *P* value of the CSF biomarker estimate (*P* < 0.01 or *P* < 0.05, for large and small respectively). Figure depicts the results of linear metabolite concentration ~ CSF biomarker model that remained significant after the correction for age and gender. Detailed results for age- and gender-corrected models are given in Additional file 1: Table S7

The association of metabolite concentrations in both plasma and CSF of AD patients with CSF biomarkers (amyloidβ(Aβ)1–42, tau and pTau-181) was evaluated using single and multiple regression modeling (age and gender-corrected, Fig. 5 and Additional file 1: Table S7). In CSF, concentrations of several aromatic (i.e., tryptophan and phenylalanine), branched-chain (i.e., isoleucine and leucine) and urea cycle amino acids (i.e., citrulline and ornithine) showed significant negative association with CSF Aβ1–42 concentrations, which remained significant after correction for age and gender. Conversely, two breakdown products of tryptophan metabolism, kynurenic acid and quinolinic acid, were significantly positively associated with CSF Aβ1–42, and tau and pTau-181, respectively (Fig. 5). For metabolites in plasma, the associations with CSF AD biomarkers were less pronounced; specifically, taurine and lysine were positively associated with pTau-181. Finally, isocitrate was found to be significantly associated with tau in plasma, and pTau-181 in both plasma and CSF.

## Discussion

Distinct systemic and CNS pathway alterations related to AD were observed in this case-control study applying a thorough stepwise metabolomics approach in concomitantly collected plasma and CSF samples from well-characterized subjects with AD and cognitively healthy controls. Amino acids were decreased, and fatty acid-oxidation metabolites and TCA cycle intermediates were increased in plasma of AD patients compared to control subjects. In their CSF, the concentrations of tryptophan pathway metabolites and creatinine were increased. Specific alterations were related to amyloid while others were associated with tau pathology and neuronal injury as measured by CSF biomarkers.

The alternative angle of viewing AD as an energy metabolism and metabolic signaling disorder has recently evolved following advancements in analytical methods and new findings on the disease’s pathophysiology [32, 33]. While decreased neuronal glucose metabolism and associated altered bioenergetics are recognized as a common feature in AD, its extent and relationships with the “core” pathological processes of AD, i.e., amyloid pathology and tau-related neurodegeneration, necessitate further investigation [1, 34–36]. Decreased glucose sensing by the brain in AD could signal a fasted state to the body and lead to compensatory activation of alternative sources to fuel the TCA cycle, such as amino and fatty acids [37]. In our study, global lower plasma concentrations of amino acids in AD patients compared to controls indeed suggest that readily available amino acids could have been used to replenish the TCA cycle [38] either by forming TCA cycle intermediates (glucogenic pathway) or by forming acetylCoA (ketogenic pathway, Fig. 2, [37]). In addition to the pool of free amino acids, fatty acid oxidation can fuel the TCA cycle via production of acetylCoA. Our results showed significantly higher concentrations of the carnitine forms of main fatty acid oxidation intermediates in plasma of AD patients compared to control subjects, i.e., long-chain acylcarnitines (LCACs, from C6 to C18) and acetylcarnitine (C2). This increase could be related to incomplete oxidation of acyl-CoA intermediates resulting in their retroconversion to acylcarnitine for the transport and release to the plasma, to avoid adverse toxic effects of their accumulation in mitochondria [39]. This fuel efflux (i.e., acetylcarnitine and LCACs) is assumed to occur when the fuel delivery exceeds energy generation capacity of the TCA cycle [39]. This is in accordance with our results showing higher concentrations of TCA cycle intermediates in plasma and CSF of AD patients. Taken together, the observed alterations in the energy metabolism hub (TCA cycle) and its anaplerotic pathways, amino acid, and fatty acid oxidation, both in plasma and CSF, imply disrupted nutrient sensing and oxidation and thus energy homeostasis in AD. These alterations appear to be of systemic origin and are reflected in CSF depending on increased BBB permeability, which is supported by the observed significant positive correlation between the CSF concentrations of these metabolites and QAlb in AD patients (Fig. 4). Moreover, several amino acids, i.e., arginine, citrulline, isoleucine, leucine, ornithine, phenylalanine, and tryptophan, were negatively associated with CSF Aβ1–42 concentrations, thus with higher cerebral amyloid burden **(**Fig. 5). This is in line with previous literature where inclusion of CSF amino acids of the one-carbon metabolism in a prediction-model improved diagnostic accuracy [20, 40]. In comparison, the associations of plasma levels of amino acids with the AD CSF biomarkers were weaker, except for lysine and taurine with CSF pTau-181.

Related to these energy metabolism alterations, creatinine was significantly increased in CSF of AD patients, negatively correlated with Qalb, and positively associated with CSF Aβ1–42. As a by-product of the high energy storage metabolite phosphocreatine [41], the observed higher concentrations of creatinine in CSF in AD may be a result of excessive phosphocreatine usage (followed by degradation) and/or disrupted creatine-phosphocreatine shuttle [42] in the conditions of inadequate glucose supply. The negative correlation of creatinine with BBB permeability (Fig. 4) implies that this process takes place in the CNS. The potential dysregulation of this process is further illustrated by the negative correlation between creatine and creatinine in both plasma and CSF (Spearman’s rho 0.46, *p* = 0.003 in plasma and − 0.33, *p* = 0.037 in CSF) in AD patients, suggesting that creatinine is produced at the expense of creatine.

Our results also highlighted the CNS-specific deregulation of the tryptophan-kynurenine pathway, with significantly higher concentrations of kynurenic acid and quinolinic acid in CSF of AD patients (Fig. 3). Both of these tryptophan metabolites were previously reported to be specifically associated with neuroinflammation in CNS diseases, including AD [43–47]. While kynurenic acid was reported as putatively neuroprotective [48], quinolinic acid is considered to be neurotoxic [47] and found to be increased in AD in model systems [48], although this was not consistently confirmed in humans [47]. Our results showed that these tryptophan catabolites were also significantly associated with core AD pathology, i.e., the putatively neuroprotective kynurenic acid was associated with lower cerebral beta-amyloid burden (higher CSF Aβ1–42 levels), whilst the neurotoxic quinolinic acid was associated with increased tau hyperphosphorylation and neuronal injury. Along with our results, previous work [49, 50] suggested the tryptophan pathway to be implicated in cerebral AD pathology and might be a possible target for disease modifying interventions.

Importantly, exploratory analysis in our study indicates more significant metabolic alterations in female AD subjects. The female susceptibility to AD has been highlighted in a very recent study [51], although the underlying mechanisms of how sex modifies AD risk are poorly understood. Different findings suggest that the profound age-related metabolic and hormonal changes in female (i.e., estrogen loss) exacerbate the peripheral and brain insulin signaling dysfunction leading to reduced glucose metabolism [52, 53]. In our study, age was slightly unbalanced between the AD patients and controls and we observed correlations of several metabolites with age. However, correction for age did not significantly change the observed difference between AD patients and control subjects, except for the acylcarnitines C14, C16, and cis-aconitate in plasma and kynurenic acid in CSF (Additional file 1: Table S8). Furthermore, the presence of the ApoE4 allele did not influence our observations (no interaction effect) as evaluated using ANOVA, except for creatinine in plasma (*P*_interaction_ = 0.02) and asparagine in CSF (*P*_interaction_ = 0.005, Additional file 1: Table S9). No difference was observed in fatty acid oxidation among individuals with different APOE genotype.

In the present study, the quantitative metabolite data acquired in paired plasma and CSF samples combined with clinical diagnosis criteria, AD CSF biomarker data, and clinical metadata allowed us to identify and quantify metabolic alterations in AD and associate them with distinct AD pathologies (amyloid pathology (Aβ1–42), neuronal injury (tau), and tau hyperphosphorylation (pTau-181)), whilst deriving information on the most likely origin of these alterations (systemic or CNS). However, it is possible that the observed metabolites are derived elsewhere, such as in the gut microbiota, which is of particular importance for tryptophan catabolism that is regulated via a highly interconnected loop involving gut microbiota [54].

To our knowledge, only one other metabolomics-led study in AD reported on both plasma and CSF [18] but relied only on untargeted discovery approach and relative comparisons without targeted quantification (i.e., validation). Although the relatively small sample size and the selection of subjects with both the clinical presentation and the presence of AD pathology (as indicated by CSF biomarkers) may be considered as limitations of this study, its quantitative character and paired investigation of plasma and CSF samples represent its asset when compared to large and heterogenous multicentric studies. Independent, larger cohort studies would allow for validation of these findings and further addressing relationships with factors such as gender and APOE genotype. Furthermore, the inclusion of other groups, in particular of subjects with normal cognition and an AD CSF biomarker profile, i.e., with preclinical AD, would enable the verification of early presence of the observed metabolic dysregulations. In a longitudinal setting, the relation between the energy metabolism alterations observed in this study and the known reduced glucose metabolism in the presymptomatic stages of AD, as well as its evolution with disease progression can be studied. This would allow for the recommendation of a new set of potentially powerful small molecule biomarkers for AD diagnosis and, more importantly, the identification of a potential target pathway(s) for prevention interventions.

## Conclusion

This metabolomics study performed using paired plasma and CSF samples from two well-defined groups highlights dysregulated systemic energy metabolism in AD and CNS-specific tryptophan pathway and creatinine alterations. In plasma of AD patients, we observed higher concentrations of TCA cycle intermediates and long-chain acylcarnitines, and lower concentrations of amino acids. These alterations appear to be of systemic origin and are mirrored in the CNS as a function of BBB permeability. The associations of specific amino acid creatinine in CSF with CSF Aβ1–42 suggest their involvement in amyloid pathology. Furthermore, our findings strongly suggest that tryptophan pathway alteration in AD is CNS-specific resulting in significantly higher concentrations of the neuroprotective kynurenic acid and neurotoxic quinolinic acid in CSF. The quantified tryptophan pathway catabolites appear to be closely related with core AD pathology, i.e., amyloid accumulation and tau-related neurodegeneration. Our study demonstrates the translational potential of the pathway-oriented quantitative approach to assess in-depth systemic and CNS metabolic defects which are part of the AD pathophysiology and represent possible targets for new therapeutic interventions.

## Supplementary information

**Additional file 1.** **Table S1.** List of metabolites whose levels were identified as significantly altered in plasma (A) and in CSF (B) of AD patients by untargeted metabolic profiling. Metabolite identities were confirmed by MS/MS data matching against standard spectral library. **Table S2.** List of additional metabolites whose levels were identified as significantly altered in plasma (A) and in CSF (B) of AD patients by an extended, highly specific and sensitive, multiple pathway targeted analysis in MRM (multiple reaction monitoring) mode. **Table S3.** Results of pathway topology analysis using either KEGG or SMPDB as a background knowledge database. Metabolite Set Enrichment Analysis (MSEA) and pathway impact analysis are described in Materials and Methods. The significantly enriched and most relevant pathways in AD as reflected in plasma are listed in Table A) and as reflected in CSF in Table B). **Table S4.** Analytical data describing the metabolite-specific information (i.e., internal standards, transitions, etc.) used for absolute quantification of metabolites implicated in selected relevant pathway. **Table S5.** Concentration ranges and group differences as a result of absolute quantification analysis of metabolites implicated in selected relevant pathways, Table A) in plasma and Table B) in CSF. Concentration distributions, per group (CTL vs. AD) are presented in Figs. 2 and 3 in the main manuscript. **Table S6.** Results of correlation analysis between QAlb and measured metabolites concentration in CSF of control subjects and AD patients. Correlation results are presented in Fig. 4 in the main manuscript. **Table S7.** Results of association analysis of metabolite concentrations in plasma (Table A) and in CSF (Table B) with AD CSF biomarkers Beta amyloid, Tau and pTau assessed using multiple linear regression. **Table S8.** Results of the association between the metabolite concentration with age and the linear regression analysis correcting the AD vs control group effect for the confounding effect of age in plasma (Table A) and in CSF (Table B). **Table S9.** Results of the ANOVA analysis for the interaction between the AD effect (group) and ApoE4 effect, Table A) in plasma and Table B) in CSF. **Figure S1.** Sex-related significant differences at the metabolite level, measured in plasma a) and b), and measured in CSF c) and d).

**Additional file 2.** Supplemental Methods.

## Data Availability

All concentration data generated during this study are included in this published article and its supplementary information files. The participant metadata is available upon reasonable request.

## Abbreviations

(Aβ)1–42: Amyloidβ1–42
AD: Alzheimer’s disease
BBB: Blood-brain barrier
CSF: Cerebrospinal fluid
CNS: Central nervous system
TCA: Tricarboxylic acid
Qalb: CSF/serum albumin ratio
