# Supplementary material for: Systemic and central nervous system metabolic alterations in Alzheimer’s disease
Source: Alzheimers Res Ther. 2019 Nov 28;11:93. doi: 10.1186/s13195-019-0551-7 (PMC6883620; doi:10.1186/s13195-019-0551-7)
Supplement: Supplementary file 1 — Additional file 1. Table S1. List of metabolites whose levels were identified as significantly altered in plasma (A) and in CSF (B) of AD patients by untargeted metabolic profiling. Metabolite identities were confirmed by MS/MS data matching against standard spectral library. Table S2. List of additional metabolites whose levels were identified as significantly altered in plasma (A) and in CSF (B) of AD patients by an extended, highly specific and sensitive, multiple pathway targeted analysis in MRM (multiple reaction monitoring) mode. Table S3. Results of pathway topology analysis using either KEGG or SMPDB as a background knowledge database. Metabolite Set Enrichment Analysis (MSEA) and pathway impact analysis are described in Materials and Methods. The significantly enriched and most relevant pathways in AD as reflected in plasma are listed in Table A) and as reflected in CSF in Table B). Table S4. Analytical data describing the metabolite-specific information (i.e., internal standards, transitions, etc.) used for absolute quantification of metabolites implicated in selected relevant pathway. Table S5. Concentration ranges and group differences as a result of absolute quantification analysis of metabolites implicated in selected relevant pathways, Table A) in plasma and Table B) in CSF. Concentration distributions, per group (CTL vs. AD) are presented in Figs. 2 and 3 in the main manuscript. Table S6. Results of correlation analysis between QAlb and measured metabolites concentration in CSF of control subjects and AD patients. Correlation results are presented in Fig. 4 in the main manuscript. Table S7. Results of association analysis of metabolite concentrations in plasma (Table A) and in CSF (Table B) with AD CSF biomarkers Beta amyloid, Tau and pTau assessed using multiple linear regression. Table S8. Results of the association between the metabolite concentration with age and the linear regression analysis correcting the AD vs control group effect for the con [file 13195_2019_551_MOESM1_ESM.docx]

**Supplemental Information for**

Systemic and central nervous system metabolic alterations in Alzheimer’s disease

Vera van der Velpen^1,6^, Tony Teav^1^, Héctor Gallart-Ayala^1^, Florence Mehl^1^, Ioana Konz^1^, Christopher Clark^6^, Aikaterini Oikonomidi^2^, Gwendoline Peyratout^2^, Hugues Henry^3^, Mauro Delorenzi^4,5^, Julijana Ivanisevic^1*^ and Julius Popp^2, 6*^

**Table S1**. List of metabolites whose levels were identified as significantly altered in plasma (A) and in CSF (B) of AD patients by untargeted metabolic profiling. Metabolite identities were confirmed by MS/MS data matching against standard spectral library.

1. **Plasma**

| Measured *m/z* | | Putative ID | Mass Error (ppm) | RT | Q-value | Fold change |
| --- | --- | --- | --- | --- | --- | --- |
| [M+H]^+^: | 156.0765 | Histidine | 5 | 10.13 | 0.016 | -1.12 |
| [M+H]^+^: | 175.0859 | Indole-acetamide | 7 | 5.73 | 0.017 | -1.15 |
| [M+H]^+^: | 265.1175 | N-Phenylacetyl-L-glutamine | 5 | 4.53 | 0.024 | 1.59 |
| [M+H]^+^: | 130.0496 | Pyroglutamic acid | 6 | 9.05 | 0.025 | -1.08 |
| [M+H]^+^: | 398.3246 | Hexadecenoyl carnitine | 6 | 3.42 | 0.029 | 1.30 |
| [M+H]^+^: | 205.0968 | Tryptophan | 4 | 7.01 | 0.032 | -1.17 |
| [M+H]^+^: | 372.3088 | Tetradecanoylcarnitine | 7 | 3.56 | 0.036 | 1.29 |
| [M+H]^+^: | 370.2936 | Tetradecenoylcarnitine | 6 | 3.54 | 0.038 | 1.38 |
| [M+H]^+^: | 414.3192 | N-stearoyl glutamic acid | 7 | 5.01 | 0.041 | 1.24 |
| [M+H]^+^: | 520.3395 | LysoPC (18:2) | 1 | 5.98 | 0.043 | -1.23 |
| [M+H]^+^: | 147.0763 | Glutamine | 4 | 9.05 | 0.047 | -1.06 |
| [M+H]^+^: | 245.0762 | Uridine / Pseudouridine | 5 | 4.90 | 0.055 | -1.17 |
| [M+H]^+^: | 147.1126 | Lysine | 5 | 10.35 | 0.055 | -1.09 |
| [M+H]^+^: | 269.1238 | Hydroxyprolyl-Histidine | 4 | 8.95 | 0.060 | -1.39 |
| [M+H]^+^: | 282.1187 | Methyladenosine | 5 | 7.59 | 0.060 | 1.09 |
| [M+H]^+^: | 595.3465 | Urobilin | 5 | 5.77 | 0.070 | 4.47 |
| [M+H]^+^: | 162.1133 | Carnitine | 2 | 7.57 | 0.075 | -1.07 |
| [M+H]^+^: | 166.0858 | Phenylalanine | 6 | 7.00 | 0.076 | -1.11 |
| [M+H]^+^: | 130.0859 | Pipecolic acid | 7 | 10.35 | 0.084 | -1.09 |
| [M+H]^+^: | 217.1290 | N-Acetyl-Arginine | 5 | 8.38 | 0.084 | -1.24 |
| [M+H]^+^: | 597.3616 | Urobilinogen | 6 | 1.39 | 0.086 | 4.51 |
| [M+H]^+^: | 502.2909 | LysoPE(20:4) | 5 | 6.08 | 0.086 | 1.19 |
| [M+H]^+^: | 344.2780 | Dodecanoylcarnitine | 6 | 3.76 | 0.094 | 1.31 |

Q-value, FDR-corrected P-value from Mann-Whitney test; ppm, parts per million.

1. **CSF**

| Measured *m/z* | | Putative ID | Mass Error (ppm) | RT | Q-value | Fold change |
| --- | --- | --- | --- | --- | --- | --- |
| [M+H]^+^: | 153.0406 | Xanthine | 4 | 5.61 | 8.06E-05 | 1.19 |
| [M+H]^+^: | 310.1128 | Acetylneuraminic acid | 3 | 9.58 | 0.000147 | 1.23 |
| [M+H]^+^: | 114.0673 | Creatinine | 5 | 4.80 | 0.000459 | 1.05 |
| [M+H]^+^: | 205.1550 | Hydroxy-trimethyllysine | 1 | 10.16 | 0.00052 | 1.19 |
| [M+H]^+^: | 203.1506 | Dimethylarginine | 1 | 9.54 | 0.001696 | 1.14 |
| [M+H]^+^: | 298.1144 | Methylguanosine | 2 | 5.71 | 0.003719 | 1.17 |
| [M+H]^+^: | 241.1295 | Homocarnosine | 2 | 10.42 | 0.005185 | -1.50 |
| [M+H]^+^: | 189.1601 | Trimethyllysine | 1 | 9.96 | 0.008553 | 1.12 |
| [M+H]^+^: | 265.1181 | Phenylacetyl-L-glutamine | 3 | 4.50 | 0.012464 | 1.84 |
| [M+H]^+^: | 595.3472 | Urobilin | 4 | 5.73 | 0.041055 | 2.38 |
| [M+H]^+^: | 290.1595 | Methylglutarylcarnitine | 3 | 6.99 | 0.041169 | 1.21 |
| [M+H]^+^: | 312.1301 | Dimethylguanosine | 2 | 5.70 | 0.059944 | 1.11 |
| [M+Na]^+^: | 203.0528 | Myoinositol / Glucose | 2 | 9.40 | 0.061148 | -1.09 |

Q-value, FDR-corrected P-value from Mann-Whitney test; ppm, parts per million.

**Table S2**. List of additional metabolites whose levels were identified as significantly altered in plasma (A) and in CSF (B) of AD patients by an extended, highly specific and sensitive, multiple pathway targeted analysis in MRM (multiple reaction monitoring) mode.

**A) Plasma**

| Metabolite | Fold change | P-value (U-test) | FDR-adjusted P-value |
| --- | --- | --- | --- |
| Uridine | -1.30 | 0.00001 | 0.003 |
| Tryptophan | -1.18 | 0.0001 | 0.06 |
| Kynurenate | -1.27 | 0.001 | 0.11 |
| Hydroxypyruvate / 3-hydroxybutanoate | 2.28 | 0.001 | 0.11 |
| Propionylcarnitine | -1.18 | 0.002 | 0.28 |
| Spermine | -1.14 | 0.002 | 0.10 |
| N-methyl-d-aspartic acid | -1.12 | 0.002 | 0.06 |
| Histidine | -1.10 | 0.002 | 0.13 |
| 1-methylnicotinamide | -1.27 | 0.002 | 0.18 |
| Carnitine | -1.16 | 0.002 | 0.06 |
| 2,5-dihydroxybenzoate | -1.44 | 0.003 | 0.13 |
| Methyl-l-histidine | -1.24 | 0.003 | 0.15 |
| Sucrose | 1.31 | 0.01 | 0.37 |
| Homocitrulline | -1.26 | 0.01 | 0.13 |
| Kynurenic acid | -1.27 | 0.01 | 0.11 |
| Benzoate | -1.46 | 0.01 | 0.14 |
| Tetradecanoylcarnitine | 1.28 | 0.01 | 0.06 |
| Phenylalanine | -1.11 | 0.01 | 0.13 |
| Deoxyribose2 | -1.13 | 0.01 | 0.26 |
| Glucuronate | 1.13 | 0.01 | 0.37 |
| Valine | -1.10 | 0.02 | 0.16 |
| Leucine | -1.11 | 0.02 | 0.25 |
| Ferulate | -1.39 | 0.02 | 0.09 |
| 2,4-dihydroxypyrimidine-5-carboxylic acid | -1.12 | 0.03 | 0.20 |
| N-acetylserine | -1.10 | 0.03 | 0.26 |
| Lysine | -1.08 | 0.03 | 0.16 |
| Pantothenic acid | -1.24 | 0.03 | 0.24 |
| Mercaptopyruvate | -1.27 | 0.03 | 0.16 |
| Thiamine | -1.65 | 0.03 | 0.24 |
| Lauroylcarnitine | 1.28 | 0.04 | 0.17 |
| N-alpha-acetyl-l-asparagine | 1.44 | 0.04 | 0.24 |
| Glutamine | -1.09 | 0.04 | 0.13 |
| Methionine | -1.07 | 0.04 | 0.37 |
| Ornithine | -1.11 | 0.04 | 0.37 |
| Trimethylamine | 1.05 | 0.04 | 0.23 |
| Mesoxalate | 1.07 | 0.04 | 0.55 |
| Methyl indole-3-acetate | -1.32 | 0.04 | 0.25 |
| (2r,3r)-(-)-2,3-butanediol | 1.11 | 0.05 | 0.27 |
| Raffinose | 1.32 | 0.05 | 0.44 |

Data represented as the mean fold change (FC), and P-value using Mann-Whitney U-test, the adjusted P-value is FDR corrected.

1. **CSF**

| Metabolite | FC | P-value (U-test) | FDR-adjusted P-value |
| --- | --- | --- | --- |
| Glucuronate | 1.31 | 0.000003 | 0.001 |
| N-acetylmannosamine peak1 | 1.17 | 0.000004 | 0.001 |
| Creatinine | 1.08 | 0.00001 | 0.01 |
| Palatinose | 1.40 | 0.00002 | 0.001 |
| Xanthine | 1.21 | 0.0001 | 0.001 |
| Glycerate | 1.15 | 0.0001 | 0.01 |
| Glucose 6-phosphate | 1.18 | 0.0002 | 0.01 |
| 3-hydroxyanthranilate | 1.20 | 0.0003 | 0.002 |
| Xanthosine | 1.25 | 0.001 | 0.01 |
| N-acetyl-d-glucosamine | 1.21 | 0.001 | 0.01 |
| Tetradecanoylcarnitine | 1.40 | 0.001 | 0.03 |
| 3-hydroxymethylglutarate | 1.17 | 0.001 | 0.01 |
| Nepsilon,nepsilon,nepsilon-trimethyllysine | 1.11 | 0.005 | 0.04 |
| S-(5-adenosyl)-l-homocysteine | 1.58 | 0.01 | 0.05 |
| 4-hydroxy-2-quinolinecarboxylic acid | 1.40 | 0.01 | 0.03 |
| Mannitol | 1.11 | 0.01 | 0.11 |
| N-alpha-acetyl-l-asparagine | 1.20 | 0.01 | 0.03 |
| Agmatine sulfate | 1.28 | 0.01 | 0.09 |
| 4-acetamidobutanoate | 1.17 | 0.01 | 0.10 |
| Melibiose | 1.30 | 0.01 | 0.04 |
| Melanin | -1.31 | 0.01 | 0.10 |
| Sorbitol | 1.12 | 0.01 | 0.11 |
| Cortisol | 1.32 | 0.01 | 0.04 |
| Salsolinol | 1.15 | 0.01 | 0.06 |
| N-acetylserine | 1.14 | 0.01 | 0.09 |
| Glucosamine 6-sulfate | 1.20 | 0.02 | 0.13 |
| Lysine | -1.12 | 0.02 | 0.25 |
| Nicotinamide | -1.35 | 0.02 | 0.32 |
| 5-methylthioadenosine | -1.12 | 0.02 | 0.22 |
| Lactose | -1.24 | 0.02 | 0.79 |
| 3-hydroxykynurenine | -1.05 | 0.03 | 0.41 |
| N-acetylmannosamine peak2 | 1.13 | 0.03 | 0.22 |
| Oxalate | 1.03 | 0.03 | 0.26 |
| Pantothenic acid | -1.16 | 0.03 | 0.37 |
| Adenine | -1.20 | 0.04 | 0.14 |
| Mesoxalate | 1.27 | 0.04 | 0.28 |
| Spermine | 1.05 | 0.04 | 0.38 |
| Leucine | -1.04 | 0.04 | 0.72 |
| Octanoylcarnitine | 1.51 | 0.04 | 0.27 |
| Norleucine | -1.04 | 0.04 | 0.75 |
| N,N-dimethyl-arginine | 1.09 | 0.04 | 0.12 |
| Palmitoylcarnitine | 1.41 | 0.05 | 0.16 |
| Saccharopine | 1.11 | 0.05 | 0.22 |

Data represented as the mean fold change (FC), and P-value using Mann-Whitney U-test, the adjusted P-value is FDR corrected.

**Table S3**. Results of pathway topology analysis using either KEGG or SMPDB as a background knowledge database. Metabolite Set Enrichment Analysis (MSEA) and pathway impact analysis are described in Materials and Methods. The significantly enriched and most relevant pathways in AD as reflected in plasma are listed in Table A) and as reflected in CSF in Table B).

1. **Plasma**

| Database | Pathway | Total compounds in pathway | Hits | P-value | FDR | Impact |
| --- | --- | --- | --- | --- | --- | --- |
| KEGG | Pyrimidine metabolism | 60 | 7 | 0.0001 | 0.01 | 0.064 |
|  | Synthesis and degradation of ketone bodies | 6 | 1 | 0.001 | 0.02 | 0.000 |
|  | beta-Alanine metabolism | 28 | 6 | 0.002 | 0.02 | 0.047 |
|  | Tryptophan metabolism | 79 | 6 | 0.002 | 0.02 | 0.219 |
|  | Pantothenate and CoA biosynthesis | 27 | 3 | 0.002 | 0.02 | 0.200 |
|  | Nitrogen metabolism | 39 | 9 | 0.004 | 0.03 | 0.008 |
|  | Phenylalanine, tyrosine and tryptophan biosynthesis | 27 | 3 | 0.004 | 0.03 | 0.008 |
|  | Aminoacyl-tRNA biosynthesis | 75 | 16 | 0.01 | 0.07 | 0.169 |
|  | Tyrosine metabolism | 76 | 7 | 0.01 | 0.08 | 0.238 |
|  | Fatty acid metabolism | 50 | 1 | 0.01 | 0.08 | 0.000 |
|  | Histidine metabolism | 44 | 6 | 0.02 | 0.09 | 0.181 |
|  | Biotin metabolism | 11 | 1 | 0.02 | 0.09 | 0.000 |
|  | Valine, leucine and isoleucine degradation | 40 | 4 | 0.03 | 0.13 | 0.022 |
|  | Butanoate metabolism | 40 | 5 | 0.04 | 0.14 | 0.033 |
|  | Valine, leucine and isoleucine biosynthesis | 27 | 3 | 0.04 | 0.15 | 0.040 |
| SMPDB | Pyrimidine Metabolism | 52 | 5 | 0.00003 | 0.002 | 0.096 |
|  | Fatty Acid Metabolism | 41 | 2 | 0.0004 | 0.01 | 0.000 |
|  | Fatty Acid Biosynthesis | 33 | 1 | 0.001 | 0.02 | 0.015 |
|  | Mitochondrial Beta-Oxidation of Long Chain Saturated Fatty Acids | 25 | 2 | 0.001 | 0.02 | 0.000 |
|  | Beta Oxidation of Very Long Chain Fatty Acids | 13 | 2 | 0.004 | 0.06 | 0.049 |
|  | Oxidation of Branched Chain Fatty Acids | 22 | 4 | 0.01 | 0.07 | 0.000 |
|  | Phenylacetate Metabolism | 8 | 1 | 0.01 | 0.07 | 0.000 |
|  | Histidine Metabolism | 35 | 5 | 0.01 | 0.07 | 0.559 |
|  | Beta-Alanine Metabolism | 27 | 6 | 0.01 | 0.09 | 0.000 |
|  | Biotin Metabolism | 7 | 1 | 0.02 | 0.11 | 0.000 |
|  | Carnitine Synthesis | 17 | 6 | 0.02 | 0.11 | 0.388 |
|  | Thiamine Metabolism | 9 | 1 | 0.02 | 0.11 | 0.000 |
|  | Pantothenate and CoA Biosynthesis | 19 | 1 | 0.03 | 0.13 | 0.069 |
|  | Ammonia Recycling | 27 | 7 | 0.03 | 0.13 | 0.362 |
|  | Nicotinate and Nicotinamide Metabolism | 34 | 7 | 0.03 | 0.13 | 0.008 |
|  | Catecholamine Biosynthesis | 14 | 3 | 0.04 | 0.13 | 0.667 |
|  | Spermidine and Spermine Biosynthesis | 14 | 5 | 0.04 | 0.13 | 0.086 |

KEGG, Kyoto Encyclopedia of Genes and Genomes; SMPDB, Small Molecule Pathway Database; FDR, False Discovery Rate

1. **CSF**

| Database | Pathway | Total compounds in pathway | Hits | P-value | FDR | Impact |
| --- | --- | --- | --- | --- | --- | --- |
| KEGG | Pentose and glucuronate interconversions | 53 | 1 | 0.000002 | 0.00002 | 0.087 |
|  | Ascorbate and aldarate metabolism | 45 | 1 | 0.000002 | 0.00002 | 0.033 |
|  | Starch and sucrose metabolism | 50 | 1 | 0.000002 | 0.00002 | 0.000 |
|  | Amino sugar and nucleotide sugar metabolism | 88 | 1 | 0.000002 | 0.00002 | 0.000 |
|  | Inositol phosphate metabolism | 39 | 1 | 0.000002 | 0.00002 | 0.000 |
|  | Caffeine metabolism | 21 | 1 | 0.0001 | 0.0005 | 0.031 |
|  | Tryptophan metabolism | 79 | 6 | 0.0003 | 0.002 | 0.276 |
|  | Glyoxylate and dicarboxylate metabolism | 50 | 6 | 0.001 | 0.005 | 0.250 |
|  | Pentose phosphate pathway | 32 | 3 | 0.001 | 0.005 | 0.065 |
|  | Glycerolipid metabolism | 32 | 2 | 0.001 | 0.01 | 0.028 |
|  | Purine metabolism | 92 | 8 | 0.002 | 0.01 | 0.075 |
|  | Steroid hormone biosynthesis | 99 | 1 | 0.01 | 0.02 | 0.030 |
|  | Fatty acid metabolism | 50 | 1 | 0.01 | 0.05 | 0.000 |
|  | Lysine degradation | 47 | 5 | 0.02 | 0.06 | 0.196 |
|  | Biotin metabolism | 11 | 1 | 0.04 | 0.13 | 0.000 |
|  | Pyruvate metabolism | 32 | 2 | 0.04 | 0.13 | 0.138 |
|  | Glycolysis or Gluconeogenesis | 31 | 2 | 0.04 | 0.13 | 0.000 |
|  | Glycine, serine and threonine metabolism | 48 | 9 | 0.04 | 0.13 | 0.309 |
| SMPDB | Inositol Metabolism | 29 | 1 | 0.000002 | 0.0001 | 0.000 |
|  | Starch and Sucrose Metabolism | 26 | 1 | 0.000002 | 0.0001 | 0.000 |
|  | Glycerolipid Metabolism | 24 | 2 | 0.001 | 0.02 | 0.259 |
|  | Steroidogenesis | 42 | 1 | 0.01 | 0.07 | 0.019 |
|  | Glycine and Serine Metabolism | 52 | 8 | 0.01 | 0.09 | 0.174 |
|  | Purine Metabolism | 66 | 10 | 0.01 | 0.10 | 0.081 |
|  | Spermidine and Spermine Biosynthesis | 14 | 5 | 0.02 | 0.13 | 0.086 |
|  | Betaine Metabolism | 19 | 3 | 0.02 | 0.18 | 0.232 |
|  | Tyrosine Metabolism | 57 | 9 | 0.03 | 0.18 | 0.027 |
|  | Carnitine Synthesis | 17 | 4 | 0.03 | 0.18 | 0.388 |
|  | Biotin Metabolism | 7 | 1 | 0.04 | 0.19 | 0.000 |
|  | Pyruvate Metabolism | 40 | 2 | 0.04 | 0.19 | 0.040 |
|  | Gluconeogenesis | 32 | 2 | 0.04 | 0.19 | 0.034 |
|  | Methionine Metabolism | 37 | 7 | 0.04 | 0.19 | 0.255 |

KEGG, Kyoto Encyclopedia of Genes and Genomes; SMPDB, Small Molecule Pathway Database; FDR, False Discovery Rate

**Table S4**. Analytical data describing the metabolite-specific information (i.e. internal standards, transitions, etc.) used for absolute quantification of metabolites implicated in selected relevant pathways.

| Metabolites | Precursor Ion (*m/z*) | Assignation | Product Ion (*quantifier transition) | Collision Energy (eV) | Ret. Time (min) | Metabolic Pathway |
| --- | --- | --- | --- | --- | --- | --- |
| *Negative mode* | | | | | | |
| Aspartate | 132.0 | [M-H]^-^ | 115, 88* | 8, 12 | 7.191 | Amino acids |
| Aspartate (13C4; 15N) | 137.1 | [M-H]^-^ | 115, 107, 90*, 88, 77 | 36, 8, 12, 8, 12 | 7.191 | Amino acids |
| Glutamate | 146.0 | [M-H]^-^ | 128*, 102 | 8, 12 | 6.988 | Amino acids |
| Glutamate (13C5; 15N) | 152.1 | [M-H]^-^ | 134*, 107 | 8, 12 | 6.988 | Amino acids |
| Glutarate | 131.0 | [M-H]^-^ | 113, 87* | 8, 12 | 7.349 | Amino acids |
| 2-oxo-glutarate / alpha-ketoglutarate | 145.0 | [M-H]^-^ | 101*, 57 | 4, 8 | 7.677 | TCA cycle |
| Alpha-ketoglutarate (13C5) | 150.1 | [M-H]^-^ | 105*, 60 | 4, 8 | 7.711 | TCA cycle |
| Acetyl-coa | 808.1 | [M-H]^-^ | 461, 408* | 40, 40 | 6.822 | TCA cycle |
| Cis-aconitate | 173.0 | [M-H]^-^ | 129* | 4 | 8.543 | TCA cycle |
| Citrate | 191.0 | [M-H]^-^ | 111*, 87 | 12, 16 | 8.643 | TCA cycle |
| Citrate (13C6) | 197.1 | [M-H]^-^ | 116*, 90 | 12, 12 | 8.62 | TCA cycle |
| Fructose | 215.1 | [M+Cl]^-^ | 215* | 5 | 5.549 | TCA cycle |
| Fructose (U-13C6) | 221.1 | [M+Cl]^-^ | 221* | 5 | 5.539 | TCA cycle |
| Fructose-6-phosphate | 259.0 | [M-H]^-^ | 97* | 16, 12 | 7.67 | TCA cycle |
| Fumarate | 115.0 | [M-H]^-^ | 71*, 27 | 4, 12 | 7.851 | TCA cycle |
| Fumarate (D4) | 117.1 | [M-H]^-^ | 100, 73*, 27 | 24, 12, 12 | 7.851 | TCA cycle |
| Glucose | 215.1 | [M+Cl]^-^ | 215* | 5 | 6 | TCA cycle |
| Glucose (U-13C6) | 221.1 | [M+Cl]^-^ | 221* | 5 | 6 | TCA cycle |
| Glucose-6-phosphate | 259.0 | [M-H]^-^ | 97* | 16, 12 | 7.923 | TCA cycle |
| Glucose-6-phosphate (U-13C6) | 265.1 | [M-H]^-^ | 97*, 79 | 12, 40 | 7.923 | TCA cycle |
| Glutaryl-coa | 880.1 | [M-H]^-^ | 533*, 408 | 40, 40 | 8.217 | TCA cycle |
| Isocitrate | 191.0 | [M-H]^-^ | 173, 155* | 8, 12 | 8.897 | TCA cycle |
| Lactate | 89.0 | [M-H]^-^ | 43, 41* | 40, 40 | 5.002 | TCA cycle |
| Lactate (3,3,3-D3) | 92.1 | [M-H]^-^ | 45*, 42 | 12, 28 | 5.002 | TCA cycle |
| Malate | 133.0 | [M-H]^-^ | 115*, 71 | 8, 16 | 7.794 | TCA cycle |
| Malate (13C4) | 137.1 | [M-H]^-^ | 119*, 74 | 8, 12 | 7.815 | TCA cycle |
| NAD+ | 662.1 | [M-H]^-^ | 540*, 328 | 28, 16 | 6.676 | TCA cycle |
| NADH | 664.1 | [M-H]^-^ | 408*, 159, 79 | 40, 40, 40 | 6.873 | TCA cycle |
| NADP | 742.1 | [M-H]^-^ | 620* | 16 | 8.068 | TCA cycle |
| NADPH | 744.1 | [M-H]^-^ | 408*, 397, 159 | 40, 40, 40 | 8.885 | TCA cycle |
| Oxoadipate | 159.1 | [M-H]^-^ | 115, 59*, 43, 41 | 8, 12, 24, 40 | 7.585 | TCA cycle |
| Phosphoenol pyruvate | 167.0 | [M-H]^-^ | 79* | 24 | 8.447 | TCA cycle |
| Phosphoenol pyruvate (2,3-13C2) | 169.0 | [M-H]^-^ | 79* | 16 | 8.447 | TCA cycle |
| Pyruvate | 87.0 | [M-H]^-^ | 87*, 43 | 4, 5 | 4.301 | TCA cycle |
| Pyruvate (D3) | 90.0 | [M-H]^-^ | 90*, 46 | 5, 4 | 4.301 | TCA cycle |
| Succinate | 117.0 | [M-H]^-^ | 99, 73* | 12, 8 | 7.474 | TCA cycle |
| Succinate (D6) | 121.1 | [M-H]^-^ | 77*, 102 | 12, 8 | 7.474 | TCA cycle |
| Trans-aconitate | 173.0 | [M-H]^-^ | 129* | 4 | 9.064 | TCA cycle |
| 3-hydroxyanthranilate | 152.0 | [M-H]^-^ | 108*, 107 | 12, 24 | 5.483 | Tryptophan metabolism |
| 3-hydroxykynurenine | 223.2 | [M-H]^-^ | 206*, 162 | 12, 4 | 5.753 | Tryptophan metabolism |
| 3-hydroxykynurenine (1,2,3-13C3; alpha-amino-15N) | 227.2 | [M-H]^-^ | 209*, 164 | 4, 12 | 5.679 | Tryptophan metabolism |
| 5-hydroxytryptophan | 219.1 | [M-H]^-^ | 144*, 132 | 20, 16 | 6.349 | Tryptophan metabolism |
| Anthranilate | 136.1 | [M-H]^-^ | 92* | 12 | 3.896 | Tryptophan metabolism |
| Anthranilate (ring-13C6) | 142.1 | [M-H]^-^ | 98* | 16 | 3.837 | Tryptophan metabolism |
| Kynurenate | 188.0 | [M-H]^-^ | 144*, 102 | 24, 32 | 3.857 | Tryptophan metabolism |
| Kynurenate (ring-D5) | 193.2 | [M-H]^-^ | 149*, 121 | 20, 36 | 3.914 | Tryptophan metabolism |
| Picolinate | 122.0 | [M-H]^-^ | 78* | 8, 30 | 7.89 | Tryptophan metabolism |
| Quinolinate | 166.0 | [M-H]^-^ | 122*, 78 | 8, 12 | 7.608 | Tryptophan metabolism |
| 4-hydroxyphenylpyruvate | 179.0 | [M-H]^-^ | 107* | 4 | 4.476 | Tyrosine metabolism |
|  |  |  |  |  |  |  |
| *Positive mode* | | | | | | |
| Acetylcarnitine (C2) | 204.1 | [M+H]^+^ | 85* | 25 | 5.9 | Acylcarnitines |
| Acetylcarnitine (C2) (D3) | 207.1 | [M+H]^+^ | 85*, 63 | 28, 16 | 6.019 | Acylcarnitines |
| Butyrylcarnitine (C4) | 232.2 | [M+H]^+^ | 85* | 25 | 4.624 | Acylcarnitines |
| Butyrylcarnitine (C4) (D3) | 235.3 | [M+H]^+^ | 85*, 63 | 24, 16 | 4.823 | Acylcarnitines |
| Carnitine (C0) | 162.1 | [M+H]^+^ | 85* | 25 | 7.075 | Acylcarnitines |
| Carnitine (C0) (D9) | 171.2 | [M+H]^+^ | 103*, 69 | 16, 20 | 7.215 | Acylcarnitines |
| Decanoylcarnitine (C10) | 316.2 | [M+H]^+^ | 85* | 25 | 3.327 | Acylcarnitines |
| Decanoylcarnitine (C10) (10,10,10-D3) | 319.3 | [M+H]^+^ | 260, 85* | 28, 12 | 3.558 | Acylcarnitines |
| Glutarylcarnitine (D3) | 279.2 | [M+H]^+^ | 85.2 | 32 | 6.82 | Acylcarnitines |
| Hexanoylcarnitine (C6) | 260.2 | [M+H]^+^ | 85* | 25 | 3.968 | Acylcarnitines |
| Hexanoylcarnitine (C6) (6, 6, 6-D3) | 263.2 | [M+H]^+^ | 85*, 204 | 24, 12 | 4.204 | Acylcarnitines |
| Hydroxyisovalerylcarnitine (D3) | 267.2 | [M+H]^+^ | 39, 43, 169 | 40, 40, 12 | 0.755 | Acylcarnitines |
| Isovalerylcarnitine (C5) | 246.2 | [M+H]^+^ | 85* | 25 | 4.261 | Acylcarnitines |
| Isovalerylcarnitine (C5) (D9) | 255.4 | [M+H]^+^ | 85* | 28 | 4.459 | Acylcarnitines |
| Lauroyl / dodecanoylcarnitine (C12) | 344.3 | [M+H]^+^ | 285, 85* | 16, 24 | 3.155 | Acylcarnitines |
| Lauroyl / dodecanoylcarnitine (C12) (D9) | 353.3 | [M+H]^+^ | 85*, 69 | 36, 24 | 3.437 | Acylcarnitines |
| Octanoylcarnitine (C8) | 288.2 | [M+H]^+^ | 85* | 25 | 3.592 | Acylcarnitines |
| Octanoylcarnitine (C8) (D3) | 291.4 | [M+H]^+^ | 229, 85* | 28, 12 | 3.814 | Acylcarnitines |
| Palmitoylcarnitine (C16) | 400.3 | [M+H]^+^ | 85* | 25 | 2.875 | Acylcarnitines |
| Palmitoylcarnitine (C16) (D3) | 403.7 | [M+H]^+^ | 85* | 25 | 2.875 | Acylcarnitines |
| Propionylcarnitine (C3) | 218.1 | [M+H]^+^ | 85* | 25 | 5.153 | Acylcarnitines |
| Propionylcarnitine (C3) (D3) | 221.2 | [M+H]^+^ | 85* | 25 | 5.153 | Acylcarnitines |
| Stearoyl / octadecanoylcarnitine (C18) | 428.4 | [M+H]^+^ | 85* | 25 | 2.601 | Acylcarnitines |
| Stearoyl / octadecanoylcarnitine (C18) (D3) | 207.1 | [M+H]^+^ | 85* | 25 | 2.601 | Acylcarnitines |
| Myristoyl / tetradecanoylcarnitine (C14) | 372.3 | [M+H]^+^ | 85* | 25 | 2.908 | Acylcarnitines |
| Myristoyl / tetradecanoylcarnitine (C14) (D9) | 381.4 | [M+H]^+^ | 85*, 69 | 28, 24 | 3.289 | Acylcarnitines |
| 4-aminobutanoate (gamma-aminobutyrate / gaba) | 104.1 | [M+H]^+^ | 87, 45* | 8, 24 | 6.876 | Amino acids |
| Alanine | 90.1 | [M+H]^+^ | 44* | 12 | 8.043 | Amino acids |
| Alanine (13C3; 15N) | 94.1 | [M+H]^+^ | 94* | 0 | 8.043 | Amino acids |
| Alpha-aminoadipate | 162.1 | [M+H]^+^ | 98*, 55 | 16, 32 | 8.406 | Amino acids |
| Anserine | 241.0 | [M+H]^+^ | 109*, 96 | 28, 40 | 10.845 | Amino acids |
| Arginine | 175.1 | [M+H]^+^ | 70*, 60 | 28, 16 | 10.547 | Amino acids |
| Arginine (13C6; 15N4) | 185.1 | [M+H]^+^ | 122, 75* | 28, 12 | 10.677 | Amino acids |
| Asparagine | 133.1 | [M+H]^+^ | 87, 74* | 20, 10 | 9.082 | Amino acids |
| Asparagine (13C4) | 137.1 | [M+H]^+^ | 76*, 73 | 10, 10 | 9.082 | Amino acids |
| Beta-alanine | 90.1 | [M+H]^+^ | 72, 30* | 10, 20 | 7.719 | Amino acids |
| Carnosine | 227.1 | [M+H]^+^ | 156, 110* | 16, 24 | 10.923 | Amino acids |
| Citrulline | 176.1 | [M+H]^+^ | 159, 70* | 8, 28 | 9.265 | Amino acids |
| Citrulline (UREIDO-13C) | 199.1 | [M+H]^+^ | 154*, 138 | 12, 8 | 9.334 | Amino acids |
| Creatinine | 114.1 | [M+H]^+^ | 86, 44* | 12, 20 | 4.73 | Amino acids |
| Creatinine (D3) | 117.1 | [M+H]^+^ | 43* | 40 | 4.6 | Amino acids |
| Cystine | 241.0 | [M+H]^+^ | 120, 74* | 23, 32 | 11.678 | Amino acids |
| Cystine (13C6; 15N2) | 249.3 | [M+H]^+^ | 156, 124, 77* | 32, 12, 23 | 11.678 | Amino acids |
| Glutamine | 147.1 | [M+H]^+^ | 130, 84* | 10, 20 | 8.863 | Amino acids |
| Glycine | 76.0 | [M+H]^+^ | 30 | 40 | 8.46 | Amino acids |
| Glycine (13C2; 15N) | 79.0 | [M+H]^+^ | 79, 33, 30* | 0, 40, 40 | 8.46 | Amino acids |
| Guanidinoacetate | 118.1 | [M+H]^+^ | 72, 43* | 16, 40 | 8.079 | Amino acids |
| Histidine | 156.1 | [M+H]^+^ | 110*, 83 | 16, 28 | 10.764 | Amino acids |
| Histidine (13C6; 15N3) | 165.1 | [M+H]^+^ | 118*, 89 | 16, 28 | 10.764 | Amino acids |
| Homocitrulline | 190.1 | [M+H]^+^ | 144*, 101 | 10, 20 | 9.091 | Amino acids |
| Isoleucine | 132.1 | [M+H]^+^ | 86*, 44 | 8, 24 | 6.478 | Amino acids |
| Isoleucine (13C6; 15N) | 139.1 | [M+H]^+^ | 92*, 74 | 8, 16 | 6.478 | Amino acids |
| Leucine | 132.1 | [M+H]^+^ | 86*, 44 | 8, 24 | 6.234 | Amino acids |
| Leucine (13C6; 15N) | 139.1 | [M+H]^+^ | 92*, 74 | 8, 16 | 6.234 | Amino acids |
| Lysine | 147.1 | [M+H]^+^ | 130, 84* | 8, 16 | 10.821 | Amino acids |
| Lysine (13C6; 15N2) | 155.1 | [M+H]^+^ | 137, 90* | 20, 8 | 10.947 | Amino acids |
| Methionine | 150.1 | [M+H]^+^ | 104, 56* | 8, 16 | 6.752 | Amino acids |
| Methionine (13C5; 15N) | 156.1 | [M+H]^+^ | 63, 60* | 16, 16 | 6.752 | Amino acids |
| Ornithine | 133.1 | [M+H]^+^ | 116, 70* | 8, 24 | 10.893 | Amino acids |
| Ornithine (15N2) | 135.1 | [M+H]^+^ | 71*, 70 | 24, 24 | 10.893 | Amino acids |
| Phenylalanine | 166.1 | [M+H]^+^ | 120*, 103 | 12, 32 | 6.185 | Amino acids |
| Phenylalanine (13C9; 15N) | 176.1 | [M+H]^+^ | 129*, 111 | 12, 32 | 6.185 | Amino acids |
| Pipecolate | 130.1 | [M+H]^+^ | 84*, 56 | 16, 36 | 7.241 | Amino acids |
| Proline | 116.1 | [M+H]^+^ | 70*, 43 | 20, 36 | 7.084 | Amino acids |
| Proline (13C5; 15N) | 122.1 | [M+H]^+^ | 75* | 28 | 7.138 | Amino acids |
| Sarcosine | 90.1 | [M+H]^+^ | 44* | 12 | 7.672 | Amino acids |
| Serine | 106.1 | [M+H]^+^ | 60*, 42 | 10, 40 | 8.94 | Amino acids |
| Serine (13C3; 15N) | 110.1 | [M+H]^+^ | 63* | 24, 24 | 8.94 | Amino acids |
| Taurine | 126.0 | [M+H]^+^ | 108*, 65 | 10, 40 | 6.998 | Amino acids |
| Taurine (1,2-13C2) | 155.0 | [M+H]^+^ | 136, 108* | 10, 12 | 6.998 | Amino acids |
| Threonine | 120.1 | [M+H]^+^ | 74*, 56 | 9, 20 | 8.405 | Amino acids |
| Threonine (13C4; 15N) | 125.1 | [M+H]^+^ | 78*, 60 | 9, 20 | 8.405 | Amino acids |
| Trans-4-hydroxyproline | 132.1 | [M+H]^+^ | 86*, 68 | 16, 24 | 8.072 | Amino acids |
| Valine | 118.1 | [M+H]^+^ | 72*, 55 | 10, 20 | 7.062 | Amino acids |
| Valine (13C5; 15N) | 124.1 | [M+H]^+^ | 77*, 59 | 10, 20 | 7.062 | Amino acids |
| 2-aminophenol | 110.1 | [M+H]^+^ | 92*, 65 | 20, 16 | 0.838 | Tryptophan metabolism |
| 6-hydroxymelatonin | 249.1 | [M+H]^+^ | 190*, 158, 130 | 16, 28, 40 | 1.1 | Tryptophan metabolism |
| Kynurenine | 209.1 | [M+H]^+^ | 192*, 94 | 8, 12 | 6.254 | Tryptophan metabolism |
| Kynurenine (ring-D4, 3,3-D2) | 215.2 | [M+H]^+^ | 198*, 98 | 20, 8 | 6.254 | Tryptophan metabolism |
| N-acetylserotonin | 219.1 | [M+H]^+^ | 160*, 115 | 16, 40 | 1.127 | Tryptophan metabolism |
| Serotonin | 177.1 | [M+H]^+^ | 160, 115* | 16, 32 | 5.374 | Tryptophan metabolism |
| Xanthurenate | 206.0 | [M+H]^+^ | 188, 160* | 20, 12 | 4.5 | Tryptophan metabolism |
| Tryptophan | 205.1 | [M+H]^+^ | 188*, 146 | 8, 20 | 6.195 | Tryptophan metabolism / Amino acids |
| Tryptophan (15N2) | 207.2 | [M+H]^+^ | 189*, 147 | 8, 16 | 6.46 | Tryptophan metabolism / Amino acids |

**Table S5**. Concentration ranges and group differences as a result of absolute quantification analysis of metabolites implicated in selected relevant pathways, Table A) in plasma and Table B) in CSF. Concentration distributions, per group (CTL vs. AD) are presented in Figure 2 and 3 in the main manuscript.

1. **Plasma**

|  | Metabolites | Conc in AD patients (µM) | Conc in control subjects (µM) | % difference | P-value | FDR |
| --- | --- | --- | --- | --- | --- | --- |
| Amino acids | Arginine | 75.99 | 73.41 | 4% | 0.518 | 0.899 |
|  | Asparagine | 42.50 | 43.37 | -2% | 0.636 | 0.922 |
|  | Citrulline | 41.41 | 41.54 | 0% | 0.954 | 0.954 |
|  | Creatinine | 75.44 | 76.20 | -1% | 0.864 | 0.954 |
|  | Glutamic acid | 191.51 | 195.84 | -2% | 0.863 | 0.954 |
|  | Glutamine | 3308.88 | 3506.38 | -6% | 0.074 | 0.258 |
|  | Glycine | 242.57 | 245.18 | -1% | 0.881 | 0.954 |
|  | Histidine | 73.12 | 80.18 | -9% | **0.014** | 0.090 |
|  | Isoleucine | 53.99 | 54.67 | -1% | 0.832 | 0.954 |
|  | Leucine | 110.49 | 118.72 | -7% | 0.155 | 0.377 |
|  | Lysine | 186.46 | 202.41 | -8% | **0.032** | 0.128 |
|  | Methionine | 22.46 | 22.98 | -2% | 0.678 | 0.922 |
|  | Ornithine | 71.36 | 73.34 | -3% | 0.628 | 0.922 |
|  | Oxoadipic acid | 0.21 | 0.20 | 7% | 0.645 | 0.922 |
|  | Phenylalanine | 57.17 | 61.20 | -7% | 0.125 | 0.326 |
|  | Pipecolic acid | 1.77 | 1.82 | -3% | 0.911 | 0.954 |
|  | Proline | 180.06 | 186.63 | -4% | 0.698 | 0.922 |
|  | Serine | 101.39 | 95.52 | 6% | 0.210 | 0.441 |
|  | Taurine | 77.40 | 67.13 | 15% | 0.160 | 0.377 |
|  | Threonine | 115.81 | 116.27 | 0% | 0.938 | 0.954 |
|  | Trans-4-hydroxy-proline | 9.05 | 9.35 | -3% | 0.778 | 0.949 |
|  | Tyrosine | 65.76 | 66.83 | -2% | 0.760 | 0.949 |
|  | Valine | 212.15 | 223.37 | -5% | 0.273 | 0.547 |
| Acylcarnitines | Carnitine_C0 | 33.46 | 37.61 | -11% | **0.026** | 0.124 |
|  | Acetylcarnitine_C2 | 9.23 | 7.74 | 19% | **0.025** | 0.124 |
|  | Propionylcarnitine_C3 | 0.35 | 0.38 | -8% | 0.360 | 0.693 |
|  | Butyrylcarnitine_C4 | 0.25 | 0.25 | 1% | 0.939 | 0.954 |
|  | Isovalerylcarnitine_C5 | 0.09 | 0.10 | -9% | 0.212 | 0.441 |
|  | Hexanoylcarnitine_C6 | 0.06 | 0.04 | 31% | **0.016** | 0.090 |
|  | Octanoylcarnitine_C8 | 0.17 | 0.13 | 35% | **0.048** | 0.179 |
|  | Decanoylcarnitine_C10 | 0.32 | 0.23 | 37% | **0.029** | 0.124 |
|  | Lauroylcarnitine_C12 | 0.10 | 0.08 | 36% | **0.012** | 0.087 |
|  | Tetradecanoylcarnitine_C14 | 0.05 | 0.04 | 34% | **0.0003** | 0.014 |
|  | Palmitoylcarnitine_C16 | 0.20 | 0.18 | 14% | **0.009** | 0.080 |
|  | Stearoylcarnitine_C18 | 0.19 | 0.16 | 21% | **0.002** | 0.030 |
| TCA cycle | 2-oxo-glutarate | 9.57 | 8.78 | 9% | 0.116 | 0.319 |
|  | cis-Aconitate | 2.23 | 1.96 | 14% | **0.002** | 0.030 |
|  | Citrate | 157.74 | 134.59 | 17% | **0.002** | 0.030 |
|  | Glucose | 7482.38 | 7250.99 | 3% | 0.540 | 0.905 |
|  | Isocitrate | 4.52 | 4.03 | 12% | 0.095 | 0.298 |
|  | Lactate | 1575.25 | 1471.71 | 7% | 0.518 | 0.899 |
|  | Pyruvate | 114.82 | 117.79 | -3% | 0.814 | 0.954 |
| Tryptophan pathway | 3-hydroxy-kynurenine | 0.07 | 0.08 | -2% | 0.688 | 0.922 |
|  | Kynurenic acid | 0.05 | 0.06 | -12% | 0.099 | 0.298 |
|  | Kynurenine | 2.11 | 2.16 | -2% | 0.709 | 0.922 |
|  | Quinolinate | 0.70 | 0.65 | 7% | 0.470 | 0.873 |
|  | Tryptophan | 53.43 | 61.01 | -12% | **0.009** | 0.080 |

Conc, concentration; AD, Alzheimers disease; % difference, difference calculated with control subjects as baseline; P-value from t-test; FDR, Benjamini-Hochberg-corrected P-value.

1. **CSF**

|  | Metabolites | Conc in AD patients (µM) | Conc in control subjects (µM) | % difference | P-value | FDR |
| --- | --- | --- | --- | --- | --- | --- |
| Amino acids | Arginine | 23.43 | 23.28 | 1% | 0.863 | 0.983 |
|  | Asparagine | 7.56 | 8.13 | -7% | 0.385 | 0.667 |
|  | Citrulline | 2.89 | 2.64 | 10% | 0.220 | 0.631 |
|  | Creatinine | 87.99 | 76.23 | 15% | **0.00001** | 0.001 |
|  | Glutamine | 598.85 | 581.89 | 3% | 0.289 | 0.667 |
|  | Histidine | 14.34 | 15.79 | -9% | **0.010** | 0.226 |
|  | Isoleucine | 5.68 | 5.71 | 0% | 0.939 | 0.983 |
|  | Leucine | 14.23 | 15.14 | -6% | 0.295 | 0.667 |
|  | Lysine | 31.06 | 33.63 | -8% | **0.040** | 0.249 |
|  | Ornithine | 6.03 | 6.11 | -1% | 0.874 | 0.983 |
|  | Phenylalanine | 10.45 | 10.55 | -1% | 0.828 | 0.983 |
|  | Pipecolic acid | 0.12 | 0.11 | 10% | 0.311 | 0.667 |
|  | Proline | 1.08 | 1.20 | -10% | 0.284 | 0.667 |
|  | Serine | 30.92 | 30.97 | 0% | 0.966 | 0.983 |
|  | Taurine | 8.84 | 8.59 | 3% | 0.639 | 0.886 |
|  | Threonine | 34.77 | 33.40 | 4% | 0.356 | 0.667 |
|  | trans-4-hydroxy-Proline | 0.50 | 0.52 | -4% | 0.731 | 0.925 |
|  | Tyrosine | 10.71 | 10.90 | -2% | 0.700 | 0.912 |
|  | Valine | 18.88 | 19.50 | -3% | 0.596 | 0.884 |
| Acylcarnitines | Carnitine_C0 | 1.38 | 1.36 | 1% | 0.913 | 0.983 |
|  | Acetylcarnitine_C2 | 0.53 | 0.49 | 9% | 0.348 | 0.667 |
|  | Propionylcarnitine_C3 | 0.03 | 0.03 | 0% | 0.983 | 0.983 |
|  | Butyrylcarnitine_C4 | 0.04 | 0.03 | 8% | 0.426 | 0.703 |
|  | Isovalerylcarnitine_C5 | 1.32E-02 | 1.37E-02 | -4% | 0.690 | 0.912 |
|  | Hexanoylcarnitine_C6 | 1.15E-03 | 9.15E-04 | 25% | 0.102 | 0.366 |
|  | Octanoylcarnitine_C8 | 7.43E-04 | 4.89E-04 | 52% | 0.055 | 0.261 |
|  | Decanoylcarnitine_C10 | 9.96E-04 | 8.32E-04 | 20% | **0.041** | 0.249 |
|  | Lauroylcarnitine_C12 | 5.46E-04 | 5.30E-04 | 3% | 0.531 | 0.815 |
| TCA cycle | 2-oxo-glutarate | 0.76 | 0.67 | 13% | **0.020** | 0.249 |
|  | cis-Aconitate | 1.94 | 1.82 | 6% | 0.087 | 0.339 |
|  | Citrate | 372.61 | 331.24 | 12% | **0.036** | 0.249 |
|  | Fructose | 189.67 | 172.70 | 10% | 0.216 | 0.631 |
|  | Glucose | 4403.66 | 4475.75 | -2% | 0.626 | 0.886 |
|  | Isocitrate | 2.69 | 2.57 | 5% | 0.441 | 0.703 |
|  | Lactate | 1861.99 | 1812.97 | 3% | 0.388 | 0.667 |
|  | Malate | 0.77 | 0.67 | 14% | 0.071 | 0.303 |
|  | Pyruvate | 133.15 | 127.56 | 4% | 0.362 | 0.667 |
| Tryptophan pathway | Anthranillic acid | 0.16 | 0.17 | -1% | 0.924 | 0.983 |
|  | Kynurenic acid | 5.50E-03 | 4.26E-03 | 29% | **0.046** | 0.249 |
|  | Quinolinate | 0.02 | 0.02 | 46% | **0.040** | 0.249 |
|  | Tryptophan | 2.58 | 2.61 | -1% | 0.780 | 0.958 |

Conc, concentration; AD, Alzheimers disease; % difference, difference calculated with control subjects as baseline; P-value from t-test; FDR, Benjamini-Hochberg-corrected P-value.

**Table S6**. Results of correlation analysis between QAlb and measured metabolites concentration in CSF of control subjects and AD patients. Correlation results are presented in Figure 4 in the main manuscript.

|  |  | Controls | | | AD | | |
| --- | --- | --- | --- | --- | --- | --- | --- |
|  | Metabolites | r | Pvalue | -logP | r | Pvalue | -logP |
| Amino acids | Arginine | -0.03 | 0.86 | 0.15 | 0.08 | 0.63 | 0.45 |
|  | Asparagine | 0.34 | **0.04** | 3.13 | 0.48 | **0.004** | 5.59 |
|  | Citrulline | 0.31 | 0.07 | 2.62 | 0.44 | **0.01** | 4.78 |
|  | Creatinine | 0.18 | 0.30 | 1.21 | -0.36 | **0.03** | 3.42 |
|  | Glutamine | 0.15 | 0.40 | 0.90 | 0.39 | **0.02** | 3.92 |
|  | Histidine | -0.04 | 0.83 | 0.18 | 0.26 | 0.13 | 2.08 |
|  | Isoleucine | 0.42 | **0.01** | 4.46 | 0.66 | **0.00001** | 11.23 |
|  | Leucine | 0.52 | **0.001** | 6.60 | 0.69 | **0.000004** | 12.51 |
|  | Lysine | 0.36 | **0.03** | 3.41 | 0.45 | **0.01** | 5.00 |
|  | Ornithine | 0.27 | 0.11 | 2.16 | 0.34 | **0.04** | 3.14 |
|  | Phenylalanine | 0.32 | 0.07 | 2.73 | 0.63 | **0.0001** | 9.77 |
|  | Pipecolic acid | -0.22 | 0.21 | 1.54 | 0.14 | 0.43 | 0.85 |
|  | Proline | 0.19 | 0.28 | 1.27 | 0.60 | **0.0001** | 9.03 |
|  | Serine | 0.20 | 0.25 | 1.40 | 0.30 | 0.08 | 2.55 |
|  | Taurine | 0.03 | 0.86 | 0.15 | -0.06 | 0.73 | 0.32 |
|  | Threonine | 0.02 | 0.93 | 0.08 | 0.43 | **0.01** | 4.56 |
|  | trans-4-hydroxy-Proline | 0.14 | 0.43 | 0.84 | 0.71 | **0.000001** | 13.48 |
|  | Tyrosine | 0.26 | 0.13 | 2.01 | 0.15 | 0.38 | 0.97 |
|  | Valine | 0.48 | **0.004** | 5.65 | 0.70 | **0.000003** | 12.57 |
| Acylcarnitines | Carnitine_C0 | 0.30 | 0.08 | 2.51 | 0.56 | **0.0005** | 7.56 |
|  | Acetylcarnitine_C2 | 0.50 | **0.002** | 6.00 | 0.73 | **0.000001** | 14.31 |
|  | Propionylcarnitine_C3 | 0.49 | **0.003** | 5.88 | 0.61 | **0.0001** | 9.20 |
|  | Butyrylcarnitine_C4 | 0.42 | **0.01** | 4.48 | 0.56 | **0.0005** | 7.65 |
|  | Isovalerylcarnitine_C5 | 0.45 | **0.01** | 5.11 | 0.61 | **0.0001** | 9.23 |
|  | Hexanoylcarnitine_C6 | 0.34 | **0.05** | 3.08 | 0.49 | **0.003** | 5.98 |
|  | Octanoylcarnitine_C8 | 0.41 | **0.02** | 4.17 | 0.53 | **0.001** | 6.76 |
|  | Decanoylcarnitine_C10 | 0.51 | **0.002** | 6.31 | 0.59 | **0.0002** | 8.45 |
|  | Lauroylcarnitine_C12 | 0.52 | **0.001** | 6.55 | 0.65 | **0.00002** | 10.71 |
| TCA cycle | 2-oxo-glutarate | 0.05 | 0.79 | 0.24 | -0.12 | 0.49 | 0.70 |
|  | cis-Aconitate | -0.13 | 0.46 | 0.77 | 0.30 | 0.08 | 2.52 |
|  | Citrate | 0.29 | 0.09 | 2.46 | 0.50 | **0.002** | 6.02 |
|  | Fructose | 0.33 | 0.05 | 2.97 | 0.01 | 0.95 | 0.05 |
|  | Glucose | -0.02 | 0.91 | 0.09 | -0.13 | 0.47 | 0.76 |
|  | Isocitrate | 0.38 | **0.03** | 3.69 | 0.43 | **0.01** | 4.53 |
|  | Lactate | 0.02 | 0.93 | 0.08 | 0.30 | 0.08 | 2.55 |
|  | Malate | -0.28 | 0.10 | 2.26 | -0.03 | 0.87 | 0.13 |
|  | Pyruvate | 0.08 | 0.67 | 0.40 | 0.36 | **0.03** | 3.36 |
| Tryptophan pathway | Anthranillic acid | -0.05 | 0.76 | 0.28 | -0.13 | 0.47 | 0.76 |
|  | Kynurenic acid | 0.14 | 0.43 | 0.86 | -0.48 | **0.004** | 5.55 |
|  | Quinolinate | 0.20 | 0.24 | 1.41 | -0.01 | 0.95 | 0.06 |
|  | Tryptophan | 0.11 | 0.54 | 0.62 | 0.38 | **0.03** | 3.66 |

**Table S7**. Results of association analysis of metabolite concentrations in plasma (Table A) and in CSF (Table B) with AD CSF biomarkers Beta amyloid, Tau and pTau assessed using multiple linear regression.

1. **Plasma**

|  | Metabolites | Model | Beta amyloid | | | | Tau | | | | pTau | | | |
| --- | --- | --- | --- | --- | --- | --- | --- | --- | --- | --- | --- | --- | --- | --- |
|  |  |  | **β** | **P_β_** | **P_model_** | **β** | | **P_β_** | **P_model_** | **β** | | **P_β_** | **P_model_** |  |
| Amino acids | Arginine | Unadjusted | -2.63E-02 | 0.27 | 0.27 | 2.21E-03 | | 0.81 | 0.81 | 1.73E-01 | | 0.14 | 0.14 |  |
|  | Arginine | Age and gender adjusted | -3.29E-02 | 0.21 | 0.49 | 2.81E-03 | | 0.77 | 0.83 | 1.84E-01 | | 0.13 | 0.39 |  |
|  | Asparagine | Unadjusted | -6.99E-03 | 0.58 | 0.58 | -1.69E-03 | | 0.72 | 0.72 | 1.54E-02 | | 0.81 | 0.81 |  |
|  | Asparagine | Age and gender adjusted | -2.26E-03 | 0.87 | 0.65 | -2.18E-03 | | 0.65 | 0.61 | 1.95E-02 | | 0.76 | 0.67 |  |
|  | Citrulline | Unadjusted | -1.26E-02 | 0.41 | 0.41 | -1.30E-03 | | 0.82 | 0.82 | 5.43E-03 | | 0.94 | 0.94 |  |
|  | Citrulline | Age and gender adjusted | -2.14E-02 | 0.19 | 0.39 | -8.61E-04 | | 0.88 | 0.73 | -2.79E-03 | | 0.97 | 0.71 |  |
|  | Creatinine | Unadjusted | 1.72E-02 | 0.45 | 0.45 | 8.29E-03 | | 0.34 | 0.34 | -2.21E-02 | | 0.85 | 0.85 |  |
|  | Creatinine | Age and gender adjusted | 3.12E-02 | 0.18 | 0.08 | 6.86E-03 | | 0.41 | 0.13 | -2.80E-02 | | 0.80 | 0.21 |  |
|  | Glutamic acid | Unadjusted | 6.20E-02 | 0.64 | 0.64 | -9.01E-02 | | 0.07 | 0.07 | -6.18E-01 | | 0.35 | 0.35 |  |
|  | Glutamic acid | Age and gender adjusted | 7.87E-02 | 0.59 | 0.87 | -9.28E-02 | | 0.07 | 0.29 | -6.57E-01 | | 0.34 | 0.69 |  |
|  | Glutamine | Unadjusted | 2.40E-01 | 0.69 | 0.69 | 3.96E-01 | | 0.08 | 0.08 | 4.47E+00 | | 0.13 | 0.13 |  |
|  | Glutamine | Age and gender adjusted | 1.06E-01 | 0.87 | 0.92 | 4.11E-01 | | 0.08 | 0.30 | 4.37E+00 | | 0.15 | 0.44 |  |
|  | Glycine | Unadjusted | 8.27E-02 | 0.39 | 0.39 | 1.24E-03 | | 0.97 | 0.97 | 5.48E-01 | | 0.25 | 0.25 |  |
|  | Glycine | Age and gender adjusted | 3.40E-02 | 0.74 | 0.46 | 5.28E-03 | | 0.89 | 0.48 | 4.90E-01 | | 0.31 | 0.27 |  |
|  | Histidine | Unadjusted | 3.33E-03 | 0.85 | 0.85 | 4.26E-03 | | 0.51 | 0.51 | 9.35E-02 | | 0.27 | 0.27 |  |
|  | Histidine | Age and gender adjusted | 7.35E-03 | 0.69 | 0.93 | 4.02E-03 | | 0.55 | 0.89 | 9.90E-02 | | 0.26 | 0.64 |  |
|  | Isoleucine | Unadjusted | -2.51E-02 | 0.24 | 0.24 | -1.37E-03 | | 0.87 | 0.87 | -1.53E-03 | | 0.99 | 0.99 |  |
|  | Isoleucine | Age and gender adjusted | -1.46E-02 | 0.52 | 0.32 | -2.48E-03 | | 0.76 | 0.37 | 9.60E-03 | | 0.93 | 0.41 |  |
|  | Leucine | Unadjusted | -2.58E-02 | 0.48 | 0.48 | -7.12E-03 | | 0.61 | 0.61 | 1.75E-02 | | 0.92 | 0.92 |  |
|  | Leucine | Age and gender adjusted | -1.32E-02 | 0.74 | 0.59 | -8.71E-03 | | 0.54 | 0.53 | 2.68E-02 | | 0.88 | 0.47 |  |
|  | Lysine | Unadjusted | -5.79E-03 | 0.89 | 0.89 | 5.99E-03 | | 0.71 | 0.71 | 4.36E-01 | | **0.03** | **0.03** |  |
|  | Lysine | Age and gender adjusted | -1.18E-02 | 0.80 | 0.86 | 5.68E-03 | | 0.73 | 0.85 | 4.25E-01 | | **0.04** | 0.14 |  |
|  | Methionine | Unadjusted | -8.89E-03 | 0.34 | 0.34 | -5.04E-04 | | 0.89 | 0.89 | 8.78E-03 | | 0.85 | 0.85 |  |
|  | Methionine | Age and gender adjusted | -7.29E-03 | 0.47 | 0.54 | -8.70E-04 | | 0.81 | 0.64 | 8.13E-03 | | 0.86 | 0.62 |  |
|  | Ornithine | Unadjusted | -5.31E-02 | **0.05** | **0.05** | 2.05E-03 | | 0.85 | 0.85 | 1.06E-01 | | 0.44 | 0.44 |  |
|  | Ornithine | Age and gender adjusted | -5.39E-02 | 0.07 | 0.27 | 1.87E-03 | | 0.86 | 0.91 | 1.22E-01 | | 0.39 | 0.73 |  |
|  | Oxoadipic acid | Unadjusted | 1.46E-04 | 0.43 | 0.43 | 1.64E-07 | | 1.00 | 1.00 | -4.61E-04 | | 0.61 | 0.61 |  |
|  | Oxoadipic acid | Age and gender adjusted | 3.39E-04 | 0.06 | **0.01** | -4.52E-06 | | 0.94 | **0.05** | -1.28E-04 | | 0.88 | 0.06 |  |
|  | Phenylalanine | Unadjusted | -2.20E-03 | 0.88 | 0.88 | -4.94E-03 | | 0.39 | 0.39 | -1.60E-03 | | 0.98 | 0.98 |  |
|  | Phenylalanine | Age and gender adjusted | -7.53E-03 | 0.64 | 0.48 | -5.05E-03 | | 0.38 | 0.39 | -1.61E-02 | | 0.83 | 0.51 |  |
|  | Pipecolic acid | Unadjusted | -3.66E-03 | 0.16 | 0.16 | -1.25E-03 | | 0.21 | 0.21 | -2.45E-02 | | 0.05 | 0.05 |  |
|  | Pipecolic acid | Age and gender adjusted | -4.89E-03 | 0.08 | 0.24 | -1.26E-03 | | 0.21 | 0.45 | -2.69E-02 | | **0.04** | 0.14 |  |
|  | Proline | Unadjusted | 1.17E-02 | 0.92 | 0.92 | 4.17E-02 | | 0.34 | 0.34 | 5.79E-01 | | 0.31 | 0.31 |  |
|  | Proline | Age and gender adjusted | 6.19E-02 | 0.61 | 0.51 | 3.69E-02 | | 0.40 | 0.43 | 6.02E-01 | | 0.29 | 0.35 |  |
|  | Serine | Unadjusted | 2.16E-02 | 0.51 | 0.51 | 7.80E-03 | | 0.53 | 0.53 | 2.48E-01 | | 0.11 | 0.11 |  |
|  | Serine | Age and gender adjusted | 2.65E-02 | 0.46 | 0.89 | 7.64E-03 | | 0.55 | 0.94 | 2.50E-01 | | 0.12 | 0.48 |  |
|  | Taurine | Unadjusted | 6.74E-02 | 0.11 | 0.11 | 3.14E-03 | | 0.85 | 0.85 | 4.54E-01 | | **0.03** | **0.03** |  |
|  | Taurine | Age and gender adjusted | 4.89E-02 | 0.26 | 0.13 | 3.75E-03 | | 0.81 | 0.22 | 4.03E-01 | | **0.04** | **0.04** |  |
|  | Threonine | Unadjusted | -2.65E-02 | 0.48 | 0.48 | -7.34E-03 | | 0.61 | 0.61 | -2.83E-02 | | 0.88 | 0.88 |  |
|  | Threonine | Age and gender adjusted | 6.65E-03 | 0.86 | 0.09 | -9.18E-03 | | 0.50 | 0.07 | 2.74E-02 | | 0.88 | 0.10 |  |
|  | Trans-4-hydroxy-proline | Unadjusted | 7.62E-04 | 0.90 | 0.90 | 1.38E-03 | | 0.57 | 0.57 | 5.07E-02 | | 0.10 | 0.10 |  |
|  | Trans-4-hydroxy-proline | Age and gender adjusted | 1.62E-03 | 0.82 | 0.88 | 1.44E-03 | | 0.56 | 0.81 | 5.50E-02 | | 0.09 | 0.31 |  |
|  | Tyrosine | Unadjusted | 6.87E-03 | 0.76 | 0.76 | -6.99E-03 | | 0.42 | 0.42 | 4.51E-03 | | 0.97 | 0.97 |  |
|  | Tyrosine | Age and gender adjusted | 7.97E-03 | 0.75 | 0.97 | -7.20E-03 | | 0.42 | 0.85 | 1.51E-03 | | 0.99 | 0.98 |  |
|  | Valine | Unadjusted | -4.65E-02 | 0.47 | 0.47 | 8.40E-04 | | 0.97 | 0.97 | 3.08E-02 | | 0.92 | 0.92 |  |
|  | Valine | Age and gender adjusted | 4.21E-05 | 1.00 | 0.10 | -3.97E-03 | | 0.87 | 0.10 | 6.20E-02 | | 0.84 | 0.06 |  |
| Acylcarnitines | Carnitine_C0 | Unadjusted | -5.25E-03 | 0.57 | 0.57 | 1.91E-03 | | 0.59 | 0.59 | 5.92E-02 | | 0.19 | 0.19 |  |
|  | Carnitine_C0 | Age and gender adjusted | -6.67E-03 | 0.51 | 0.84 | 1.85E-03 | | 0.62 | 0.88 | 5.73E-02 | | 0.22 | 0.55 |  |
|  | Acetylcarnitine_C2 | Unadjusted | -1.63E-03 | 0.69 | 0.69 | -1.70E-03 | | 0.28 | 0.28 | -2.53E-02 | | 0.21 | 0.21 |  |
|  | Acetylcarnitine_C2 | Age and gender adjusted | -3.34E-03 | 0.45 | 0.64 | -1.58E-03 | | 0.32 | 0.55 | -2.64E-02 | | 0.20 | 0.47 |  |
|  | Propionylcarnitine_C3 | Unadjusted | -2.48E-04 | 0.29 | 0.29 | -3.23E-05 | | 0.72 | 0.72 | -4.05E-04 | | 0.73 | 0.73 |  |
|  | Propionylcarnitine_C3 | Age and gender adjusted | -2.49E-04 | 0.33 | 0.76 | -3.57E-05 | | 0.70 | 0.95 | -3.90E-04 | | 0.75 | 0.93 |  |
|  | Butyrylcarnitine_C4 | Unadjusted | -1.40E-04 | 0.49 | 0.49 | 4.31E-07 | | 1.00 | 1.00 | -7.74E-05 | | 0.94 | 0.94 |  |
|  | Butyrylcarnitine_C4 | Age and gender adjusted | -2.42E-04 | 0.26 | 0.29 | 3.92E-07 | | 1.00 | 0.49 | -2.95E-04 | | 0.77 | 0.49 |  |
|  | Isovalerylcarnitine_C5 | Unadjusted | -2.50E-05 | 0.51 | 0.51 | -1.28E-05 | | 0.38 | 0.38 | -1.13E-04 | | 0.55 | 0.55 |  |
|  | Isovalerylcarnitine_C5 | Age and gender adjusted | -1.30E-05 | 0.75 | 0.66 | -1.43E-05 | | 0.33 | 0.48 | -1.07E-04 | | 0.58 | 0.52 |  |
|  | Hexanoylcarnitine_C6 | Unadjusted | 2.47E-05 | 0.57 | 0.57 | -1.62E-05 | | 0.33 | 0.33 | -2.73E-04 | | 0.20 | 0.20 |  |
|  | Hexanoylcarnitine_C6 | Age and gender adjusted | 2.45E-05 | 0.61 | 0.93 | -1.64E-05 | | 0.34 | 0.77 | -2.90E-04 | | 0.19 | 0.59 |  |
|  | Octanoylcarnitine_C8 | Unadjusted | 2.11E-05 | 0.90 | 0.90 | -5.92E-05 | | 0.36 | 0.36 | -1.10E-03 | | 0.19 | 0.19 |  |
|  | Octanoylcarnitine_C8 | Age and gender adjusted | 3.46E-05 | 0.85 | 0.95 | -6.21E-05 | | 0.35 | 0.74 | -1.15E-03 | | 0.18 | 0.54 |  |
|  | Decanoylcarnitine_C10 | Unadjusted | 8.34E-06 | 0.98 | 0.98 | -9.89E-05 | | 0.37 | 0.37 | -2.01E-03 | | 0.16 | 0.16 |  |
|  | Decanoylcarnitine_C10 | Age and gender adjusted | 1.97E-05 | 0.95 | 0.93 | -1.04E-04 | | 0.35 | 0.72 | -2.13E-03 | | 0.14 | 0.46 |  |
|  | Lauroylcarnitine_C12 | Unadjusted | -1.12E-05 | 0.88 | 0.88 | -3.88E-05 | | 0.19 | 0.19 | -6.77E-04 | | 0.07 | 0.07 |  |
|  | Lauroylcarnitine_C12 | Age and gender adjusted | -3.27E-06 | 0.97 | 0.59 | -4.17E-05 | | 0.16 | 0.26 | -7.28E-04 | | 0.06 | 0.13 |  |
|  | Tetradecanoylcarnitine_C14 | Unadjusted | 1.09E-06 | 0.97 | 0.97 | -1.11E-05 | | 0.26 | 0.26 | -1.42E-04 | | 0.27 | 0.27 |  |
|  | Tetradecanoylcarnitine_C14 | Age and gender adjusted | -7.64E-07 | 0.98 | 0.45 | -1.19E-05 | | 0.23 | 0.24 | -1.67E-04 | | 0.19 | 0.21 |  |
|  | Palmitoylcarnitine_C16 | Unadjusted | -9.60E-06 | 0.88 | 0.88 | -1.32E-05 | | 0.59 | 0.59 | -9.81E-05 | | 0.76 | 0.76 |  |
|  | Palmitoylcarnitine_C16 | Age and gender adjusted | -1.15E-05 | 0.86 | 0.20 | -1.59E-05 | | 0.50 | 0.17 | -1.64E-04 | | 0.59 | 0.16 |  |
|  | Stearoylcarnitine_C18 | Unadjusted | 1.05E-05 | 0.88 | 0.88 | 2.65E-05 | | 0.33 | 0.33 | 4.17E-04 | | 0.23 | 0.23 |  |
|  | Stearoylcarnitine_C18 | Age and gender adjusted | 2.53E-05 | 0.74 | 0.74 | 2.45E-05 | | 0.38 | 0.59 | 4.14E-04 | | 0.24 | 0.39 |  |
| TCA cycle | 2-oxo-glutarate | Unadjusted | -8.76E-05 | 0.98 | 0.98 | 8.20E-04 | | 0.55 | 0.55 | 4.46E-04 | | 0.98 | 0.98 |  |
|  | 2-oxo-glutarate | Age and gender adjusted | -1.37E-03 | 0.72 | 0.53 | 9.83E-04 | | 0.48 | 0.46 | 9.34E-04 | | 0.96 | 0.62 |  |
|  | cis-Aconitate | Unadjusted | 4.26E-04 | 0.41 | 0.41 | -1.32E-04 | | 0.51 | 0.51 | -1.63E-03 | | 0.52 | 0.52 |  |
|  | cis-Aconitate | Age and gender adjusted | 8.50E-05 | 0.88 | 0.28 | -1.09E-04 | | 0.58 | 0.25 | -2.13E-03 | | 0.39 | 0.29 |  |
|  | Citrate | Unadjusted | 2.21E-02 | 0.62 | 0.62 | 3.12E-02 | | 0.06 | 0.06 | 3.78E-01 | | 0.08 | 0.08 |  |
|  | Citrate | Age and gender adjusted | -2.98E-03 | 0.95 | 0.47 | 3.29E-02 | | **0.04** | 0.08 | 3.46E-01 | | 0.11 | 0.17 |  |
|  | Glucose | Unadjusted | 5.93E-02 | 0.98 | 0.98 | -1.42E-01 | | 0.88 | 0.88 | 3.39E+00 | | 0.79 | 0.79 |  |
|  | Glucose | Age and gender adjusted | 8.78E-01 | 0.75 | 0.56 | -2.49E-01 | | 0.80 | 0.57 | 3.07E+00 | | 0.81 | 0.59 |  |
|  | Isocitrate | Unadjusted | 1.37E-03 | 0.44 | 0.44 | 1.42E-03 | | **0.03** | **0.03** | 2.33E-02 | | **0.004** | **0.004** |  |
|  | Isocitrate | Age and gender adjusted | 8.57E-04 | 0.66 | 0.61 | 1.44E-03 | | **0.03** | 0.10 | 2.20E-02 | | **0.01** | **0.02** |  |
|  | Lactate | Unadjusted | 7.69E-01 | 0.55 | 0.55 | 2.62E-01 | | 0.59 | 0.59 | 8.39E+00 | | 0.19 | 0.19 |  |
|  | Lactate | Age and gender adjusted | 8.19E-01 | 0.56 | 0.80 | 2.89E-01 | | 0.57 | 0.80 | 9.01E+00 | | 0.17 | 0.47 |  |
|  | Pyruvate | Unadjusted | 1.19E-01 | 0.15 | 0.15 | -4.30E-03 | | 0.89 | 0.89 | 4.49E-01 | | 0.28 | 0.28 |  |
|  | Pyruvate | Age and gender adjusted | 1.34E-01 | 0.13 | 0.37 | -2.60E-03 | | 0.94 | 0.83 | 4.94E-01 | | 0.24 | 0.54 |  |
| Tryptophan pathway | 3-hydroxy-kynurenine | Unadjusted | 3.34E-05 | 0.16 | 0.16 | -3.96E-06 | | 0.67 | 0.67 | -8.31E-05 | | 0.48 | 0.48 |  |
|  | 3-hydroxy-kynurenine | Age and gender adjusted | 2.39E-05 | 0.35 | 0.35 | -2.90E-06 | | 0.75 | 0.48 | -9.59E-05 | | 0.42 | 0.50 |  |
|  | Kynurenic acid | Unadjusted | 1.70E-05 | 0.47 | 0.47 | -1.10E-06 | | 0.90 | 0.90 | -8.19E-05 | | 0.46 | 0.46 |  |
|  | Kynurenic acid | Age and gender adjusted | 2.33E-05 | 0.35 | 0.40 | -1.98E-06 | | 0.83 | 0.55 | -9.60E-05 | | 0.40 | 0.51 |  |
|  | Kynurenine | Unadjusted | 8.85E-04 | 0.28 | 0.28 | -3.03E-04 | | 0.33 | 0.33 | -6.38E-03 | | 0.11 | 0.11 |  |
|  | Kynurenine | Age and gender adjusted | 6.43E-04 | 0.46 | 0.43 | -2.99E-04 | | 0.34 | 0.37 | -7.37E-03 | | 0.07 | 0.14 |  |
|  | Quinolinate | Unadjusted | 4.51E-04 | 0.30 | 0.30 | -1.56E-04 | | 0.35 | 0.35 | -2.55E-03 | | 0.24 | 0.24 |  |
|  | Quinolinate | Age and gender adjusted | 1.49E-04 | 0.74 | 0.19 | -1.39E-04 | | 0.39 | 0.14 | -3.14E-03 | | 0.13 | 0.08 |  |
|  | Tryptophan | Unadjusted | -1.41E-02 | 0.44 | 0.44 | -6.04E-03 | | 0.39 | 0.39 | 4.70E-02 | | 0.60 | 0.60 |  |
|  | Tryptophan | Age and gender adjusted | -1.69E-02 | 0.40 | 0.86 | -6.06E-03 | | 0.40 | 0.86 | 4.77E-02 | | 0.61 | 0.95 |  |

β , beta-coefficient, P_β_, P-value of beta coefficient, P_model_ , P-value of model. Unadjusted model represents Metabolite=CSF biomarker, Age and gender adjusted model represents Metabolite=CSF biomarker + age + gender.

1. **CSF**

|  | Metabolites | Model | Beta amyloid | | | Tau | | | pTau | | |
| --- | --- | --- | --- | --- | --- | --- | --- | --- | --- | --- | --- |
|  |  |  | **β** | **P_β_** | **P_model_** | **β** | **P_β_** | **P_model_** | **β** | **P_β_** | **P_model_** |
| Amino acids | Arginine | Unadjusted | -1.28E-02 | **0.03** | **0.03** | -1.46E-03 | 0.53 | 0.53 | -6.10E-03 | 0.84 | 0.84 |
|  | Arginine | Age and gender adjusted | -1.19E-02 | 0.05 | **0.04** | -1.27E-03 | 0.58 | 0.18 | 3.24E-03 | 0.91 | 0.22 |
|  | Asparagine | Unadjusted | -4.84E-03 | 0.06 | 0.06 | -1.18E-04 | 0.91 | 0.91 | 8.23E-03 | 0.52 | 0.52 |
|  | Asparagine | Age and gender adjusted | -4.07E-03 | 0.13 | 0.23 | -1.77E-04 | 0.86 | 0.56 | 1.11E-02 | 0.38 | 0.33 |
|  | Citrulline | Unadjusted | -3.18E-03 | **0.03** | **0.03** | 2.27E-04 | 0.70 | 0.70 | 5.73E-04 | 0.94 | 0.94 |
|  | Citrulline | Age and gender adjusted | -3.16E-03 | **0.05** | 0.21 | 2.10E-04 | 0.72 | 0.90 | 1.32E-03 | 0.86 | 0.89 |
|  | Creatinine | Unadjusted | 4.20E-02 | **0.02** | **0.02** | 1.11E-02 | 0.12 | 0.12 | 9.25E-02 | 0.31 | 0.31 |
|  | Creatinine | Age and gender adjusted | 3.95E-02 | **0.04** | 0.13 | 1.16E-02 | 0.10 | 0.25 | 8.40E-02 | 0.37 | 0.54 |
|  | Glutamine | Unadjusted | -1.56E-01 | 0.12 | 0.12 | 3.35E-02 | 0.38 | 0.38 | 5.37E-01 | 0.28 | 0.28 |
|  | Glutamine | Age and gender adjusted | -1.11E-01 | 0.28 | 0.15 | 2.89E-02 | 0.44 | 0.19 | 6.42E-01 | 0.18 | 0.09 |
|  | Histidine | Unadjusted | -2.71E-03 | 0.41 | 0.41 | -1.87E-03 | 0.13 | 0.13 | -2.27E-02 | 0.14 | 0.14 |
|  | Histidine | Age and gender adjusted | -2.41E-03 | 0.49 | 0.62 | -1.82E-03 | 0.15 | 0.32 | -2.04E-02 | 0.20 | 0.39 |
|  | Isoleucine | Unadjusted | -5.89E-03 | **0.01** | **0.01** | 3.35E-04 | 0.71 | 0.71 | -1.49E-04 | 0.99 | 0.99 |
|  | Isoleucine | Age and gender adjusted | -4.80E-03 | **0.03** | **0.004** | 1.65E-04 | 0.84 | **0.04** | 1.80E-03 | 0.86 | **0.02** |
|  | Leucine | Unadjusted | -1.41E-02 | **0.01** | **0.01** | 1.97E-04 | 0.93 | 0.93 | -4.22E-05 | 1.00 | 1.00 |
|  | Leucine | Age and gender adjusted | -1.14E-02 | **0.04** | **0.01** | -1.98E-04 | 0.92 | 0.06 | 5.30E-03 | 0.83 | **0.02** |
|  | Lysine | Unadjusted | -9.44E-03 | 0.18 | 0.18 | -4.48E-04 | 0.87 | 0.87 | 7.69E-03 | 0.81 | 0.81 |
|  | Lysine | Age and gender adjusted | -7.21E-03 | 0.33 | 0.44 | -6.03E-04 | 0.82 | 0.62 | 1.50E-02 | 0.64 | 0.29 |
|  | Ornithine | Unadjusted | -1.04E-02 | **0.003** | **0.003** | 3.32E-04 | 0.81 | 0.81 | 1.88E-03 | 0.92 | 0.92 |
|  | Ornithine | Age and gender adjusted | -8.84E-03 | **0.01** | **0.01** | 2.32E-04 | 0.86 | 0.20 | 7.58E-03 | 0.67 | 0.17 |
|  | Phenylalanine | Unadjusted | -6.11E-03 | **0.02** | **0.02** | -4.39E-04 | 0.67 | 0.67 | -2.90E-03 | 0.82 | 0.82 |
|  | Phenylalanine | Age and gender adjusted | -5.64E-03 | **0.03** | **0.03** | -5.96E-04 | 0.54 | 0.19 | -2.09E-03 | 0.86 | 0.06 |
|  | Pipecolic acid | Unadjusted | -1.25E-04 | 0.10 | 0.10 | -4.55E-05 | 0.11 | 0.11 | -7.74E-04 | **0.03** | **0.03** |
|  | Pipecolic acid | Age and gender adjusted | -1.48E-04 | 0.07 | 0.21 | -4.74E-05 | 0.10 | 0.28 | -8.43E-04 | **0.02** | 0.10 |
|  | Proline | Unadjusted | -6.63E-04 | 0.20 | 0.20 | -6.80E-05 | 0.73 | 0.73 | -2.89E-05 | 0.99 | 0.99 |
|  | Proline | Age and gender adjusted | -6.22E-04 | 0.26 | 0.50 | -8.35E-05 | 0.68 | 0.75 | 6.73E-05 | 0.98 | 0.67 |
|  | Serine | Unadjusted | 8.05E-04 | 0.91 | 0.91 | 6.69E-04 | 0.82 | 0.82 | 2.11E-03 | 0.95 | 0.95 |
|  | Serine | Age and gender adjusted | 2.13E-03 | 0.79 | 0.91 | 5.32E-04 | 0.86 | 0.92 | 2.93E-03 | 0.94 | 0.96 |
|  | Taurine | Unadjusted | 2.35E-03 | 0.46 | 0.46 | -6.00E-04 | 0.62 | 0.62 | -3.13E-03 | 0.82 | 0.82 |
|  | Taurine | Age and gender adjusted | 1.14E-04 | 0.97 | 0.11 | -4.35E-04 | 0.71 | 0.10 | -6.56E-03 | 0.63 | 0.19 |
|  | Threonine | Unadjusted | -1.73E-02 | 0.11 | 0.11 | -5.74E-03 | 0.16 | 0.16 | -8.46E-02 | 0.11 | 0.11 |
|  | Threonine | Age and gender adjusted | -6.11E-03 | 0.53 | **0.003** | -6.31E-03 | 0.07 | **0.001** | -6.06E-02 | 0.17 | **0.00** |
|  | trans-4-hydroxy-Proline | Unadjusted | -4.66E-04 | 0.17 | 0.17 | 1.31E-04 | 0.31 | 0.31 | 9.24E-04 | 0.58 | 0.58 |
|  | trans-4-hydroxy-Proline | Age and gender adjusted | -3.54E-04 | 0.33 | 0.42 | 1.25E-04 | 0.34 | 0.43 | 1.29E-03 | 0.44 | 0.40 |
|  | Tyrosine | Unadjusted | -2.21E-03 | 0.50 | 0.50 | -1.45E-03 | 0.24 | 0.24 | -6.00E-03 | 0.70 | 0.70 |
|  | Tyrosine | Age and gender adjusted | -2.04E-03 | 0.56 | 0.65 | -1.56E-03 | 0.21 | 0.41 | -6.49E-03 | 0.69 | 0.54 |
|  | Valine | Unadjusted | -1.26E-02 | 0.11 | 0.11 | 6.59E-04 | 0.83 | 0.83 | 1.44E-03 | 0.97 | 0.97 |
|  | Valine | Age and gender adjusted | -9.71E-03 | 0.23 | 0.08 | 1.70E-04 | 0.95 | 0.15 | 5.45E-03 | 0.88 | 0.09 |
| Acylcarnitines | Carnitine_C0 | Unadjusted | -1.03E-03 | 0.09 | 0.09 | 8.33E-05 | 0.73 | 0.73 | 9.99E-04 | 0.74 | 0.74 |
|  | Carnitine_C0 | Age and gender adjusted | -8.97E-04 | 0.17 | 0.36 | 7.07E-05 | 0.77 | 0.71 | 1.52E-03 | 0.62 | 0.50 |
|  | Acetylcarnitine_C2 | Unadjusted | -5.47E-04 | 0.10 | 0.10 | 5.28E-05 | 0.68 | 0.68 | -4.25E-05 | 0.98 | 0.98 |
|  | Acetylcarnitine_C2 | Age and gender adjusted | -4.87E-04 | 0.17 | 0.35 | 4.35E-05 | 0.74 | 0.68 | 1.53E-04 | 0.93 | 0.51 |
|  | Propionylcarnitine_C3 | Unadjusted | -3.69E-05 | 0.07 | 0.07 | 7.88E-07 | 0.92 | 0.92 | -3.15E-05 | 0.76 | 0.76 |
|  | Propionylcarnitine_C3 | Age and gender adjusted | -3.17E-05 | 0.14 | 0.24 | 1.33E-07 | 0.99 | 0.57 | -1.71E-05 | 0.87 | 0.44 |
|  | Butyrylcarnitine_C4 | Unadjusted | -1.43E-05 | 0.53 | 0.53 | 1.87E-05 | **0.02** | **0.02** | 1.74E-04 | 0.11 | 0.11 |
|  | Butyrylcarnitine_C4 | Age and gender adjusted | -2.03E-05 | 0.38 | 0.19 | 1.79E-05 | **0.03** | **0.03** | 1.55E-04 | 0.14 | 0.08 |
|  | Isovalerylcarnitine_C5 | Unadjusted | -8.41E-06 | 0.25 | 0.25 | 2.45E-06 | 0.38 | 0.38 | 1.44E-05 | 0.69 | 0.69 |
|  | Isovalerylcarnitine_C5 | Age and gender adjusted | -4.59E-06 | 0.54 | 0.13 | 2.05E-06 | 0.45 | 0.12 | 2.11E-05 | 0.54 | 0.08 |
|  | Hexanoylcarnitine_C6 | Unadjusted | -4.40E-07 | 0.66 | 0.66 | 2.57E-08 | 0.95 | 0.95 | -3.06E-06 | 0.53 | 0.53 |
|  | Hexanoylcarnitine_C6 | Age and gender adjusted | -5.01E-07 | 0.65 | 0.87 | 8.00E-09 | 0.98 | 0.92 | -3.39E-06 | 0.50 | 0.75 |
|  | Octanoylcarnitine_C8 | Unadjusted | -3.73E-07 | 0.68 | 0.68 | -4.03E-08 | 0.91 | 0.91 | -2.21E-06 | 0.61 | 0.61 |
|  | Octanoylcarnitine_C8 | Age and gender adjusted | -1.47E-07 | 0.88 | 0.84 | -5.76E-08 | 0.87 | 0.84 | -1.78E-06 | 0.69 | 0.69 |
|  | Decanoylcarnitine_C10 | Unadjusted | -5.80E-08 | 0.91 | 0.91 | -7.97E-08 | 0.70 | 0.70 | -2.22E-06 | 0.39 | 0.39 |
|  | Decanoylcarnitine_C10 | Age and gender adjusted | 5.02E-08 | 0.93 | 0.87 | -9.24E-08 | 0.66 | 0.82 | -2.15E-06 | 0.42 | 0.63 |
|  | Lauroylcarnitine_C12 | Unadjusted | -5.69E-08 | 0.60 | 0.60 | -1.91E-09 | 0.96 | 0.96 | -2.47E-07 | 0.64 | 0.64 |
|  | Lauroylcarnitine_C12 | Age and gender adjusted | -5.09E-08 | 0.66 | 0.60 | -5.84E-09 | 0.89 | 0.64 | -2.79E-07 | 0.61 | 0.52 |
| TCA cycle | 2-oxo-glutarate | Unadjusted | -6.87E-06 | 0.98 | 0.98 | -1.84E-04 | 0.06 | 0.06 | -2.24E-03 | 0.07 | 0.07 |
|  | 2-oxo-glutarate | Age and gender adjusted | -1.22E-04 | 0.66 | 0.62 | -1.80E-04 | 0.06 | 0.16 | -2.50E-03 | **0.05** | 0.15 |
|  | cis-Aconitate | Unadjusted | -2.67E-05 | 0.95 | 0.95 | -1.22E-05 | 0.95 | 0.95 | 4.18E-05 | 0.99 | 0.99 |
|  | cis-Aconitate | Age and gender adjusted | 2.60E-04 | 0.59 | 0.32 | -1.90E-05 | 0.91 | 0.36 | 7.19E-04 | 0.75 | 0.28 |
|  | Citrate | Unadjusted | -2.06E-01 | 0.14 | 0.14 | 4.81E-02 | 0.37 | 0.37 | 4.65E-02 | 0.95 | 0.95 |
|  | Citrate | Age and gender adjusted | -1.35E-01 | 0.34 | 0.05 | 3.90E-02 | 0.44 | 0.06 | 1.49E-01 | 0.82 | 0.07 |
|  | Fructose | Unadjusted | -4.74E-02 | 0.66 | 0.66 | 2.34E-02 | 0.57 | 0.57 | 4.99E-01 | 0.34 | 0.34 |
|  | Fructose | Age and gender adjusted | -3.65E-02 | 0.75 | 0.75 | 2.03E-02 | 0.62 | 0.72 | 5.10E-01 | 0.34 | 0.49 |
|  | Glucose | Unadjusted | 3.55E-01 | 0.76 | 0.76 | -4.50E-01 | 0.30 | 0.30 | -1.45E+00 | 0.79 | 0.79 |
|  | Glucose | Age and gender adjusted | 5.30E-01 | 0.67 | 0.96 | -4.55E-01 | 0.30 | 0.75 | -1.13E+00 | 0.85 | 0.97 |
|  | Isocitrate | Unadjusted | -1.68E-03 | 0.13 | 0.13 | 5.53E-04 | 0.19 | 0.19 | 1.31E-02 | **0.01** | **0.01** |
|  | Isocitrate | Age and gender adjusted | -1.22E-03 | 0.28 | 0.15 | 5.00E-04 | 0.22 | 0.13 | 1.44E-02 | **0.00** | **0.00** |
|  | Lactate | Unadjusted | -4.30E-02 | 0.91 | 0.91 | -1.97E-01 | 0.15 | 0.15 | -1.33E+00 | 0.44 | 0.44 |
|  | Lactate | Age and gender adjusted | 1.11E-01 | 0.77 | 0.29 | -2.18E-01 | 0.11 | 0.09 | -1.22E+00 | 0.46 | 0.09 |
|  | Malate | Unadjusted | -4.83E-04 | 0.23 | 0.23 | -6.58E-05 | 0.67 | 0.67 | 3.45E-04 | 0.86 | 0.86 |
|  | Malate | Age and gender adjusted | -5.96E-04 | 0.17 | 0.55 | -6.60E-05 | 0.68 | 0.95 | 2.29E-04 | 0.91 | 0.98 |
|  | Pyruvate | Unadjusted | -3.55E-02 | 0.37 | 0.37 | 1.34E-03 | 0.93 | 0.93 | 7.09E-02 | 0.72 | 0.72 |
|  | Pyruvate | Age and gender adjusted | -1.55E-02 | 0.70 | 0.17 | -8.70E-04 | 0.95 | 0.18 | 1.00E-01 | 0.60 | 0.17 |
| Tryptophan pathway | Anthranillic acid | Unadjusted | 5.13E-05 | 0.46 | 0.46 | -4.13E-05 | 0.11 | 0.11 | -7.38E-04 | **0.03** | **0.03** |
|  | Anthranillic acid | Age and gender adjusted | 7.98E-05 | 0.28 | 0.42 | -4.08E-05 | 0.12 | 0.26 | -6.90E-04 | **0.04** | 0.12 |
|  | Kynurenic acid | Unadjusted | 1.19E-05 | **0.01** | **0.01** | 8.26E-08 | 0.96 | 0.96 | 2.98E-06 | 0.90 | 0.90 |
|  | Kynurenic acid | Age and gender adjusted | 8.59E-06 | **0.05** | **0.004** | 3.08E-07 | 0.85 | **0.03** | -5.74E-06 | 0.78 | **0.02** |
|  | Quinolinate | Unadjusted | -2.81E-06 | 0.90 | 0.90 | 2.38E-05 | **0.004** | **0.004** | 2.65E-04 | **0.01** | **0.01** |
|  | Quinolinate | Age and gender adjusted | -9.94E-06 | 0.69 | 0.79 | 2.39E-05 | **0.005** | **0.03** | 2.51E-04 | **0.01** | **0.05** |
|  | Tryptophan | Unadjusted | -1.67E-03 | **0.03** | **0.03** | -1.31E-04 | 0.67 | 0.67 | -1.80E-03 | 0.64 | 0.64 |
|  | Tryptophan | Age and gender adjusted | -1.58E-03 | 0.05 | 0.12 | -1.63E-04 | 0.59 | 0.50 | -1.58E-03 | 0.68 | 0.37 |

β , beta-coefficient, P_β_, P-value of beta coefficient, P_model_ , P-value of model. Unadjusted model represents Metabolite=CSF biomarker, Age and gender adjusted model represents Metabolite=CSF biomarker + age + gender.

**Table S8**. Results of the association between the metabolite concentration with age and the linear regression analysis correcting the AD vs control group effect for the confounding effect of age in plasma (Table A) and in CSF (Table B).

1. **Plasma data**

|  | Metabolites | Metabolite conc = age | | | Metabolite conc = group | | | Metabolite conc= group + age | | | | |
| --- | --- | --- | --- | --- | --- | --- | --- | --- | --- | --- | --- | --- |
|  |  | **β_age_** | **P_β_** | **β_group_** | | **P_β_** | **β_group_** | | **P_β_** | **P_age_** | **P_model_** |  |
| Amino acids | Arginine | 0.24 | 0.35 | 2.58 | | 0.52 | 0.39 | | 0.94 | 0.49 | 0.64 |  |
|  | Asparagine | -0.01 | 0.93 | -0.87 | | 0.64 | -1.25 | | 0.60 | 0.80 | 0.87 |  |
|  | Citrulline | 0.11 | 0.48 | -0.14 | | 0.95 | -1.95 | | 0.52 | 0.34 | 0.63 |  |
|  | Creatinine | 0.41 | 0.14 | -0.76 | | 0.86 | -7.74 | | 0.16 | **0.04** | 0.12 |  |
|  | Glutamic acid | 2.06 | 0.19 | -4.33 | | 0.86 | -39.78 | | 0.20 | 0.07 | 0.19 |  |
|  | Glutamine | -11.12 | 0.11 | -197.50 | | 0.07 | -145.52 | | 0.30 | 0.55 | 0.17 |  |
|  | Glycine | 0.18 | 0.87 | -2.61 | | 0.88 | -7.10 | | 0.75 | 0.75 | 0.94 |  |
|  | Histidine | -0.26 | 0.17 | -7.06 | | **0.01** | -7.47 | | **0.04** | 0.86 | **0.05** |  |
|  | Homocystine | 0.003 | 0.92 | 0.53 | | 0.21 | 0.82 | | 0.13 | 0.39 | 0.32 |  |
|  | Isoleucine | 0.10 | 0.62 | -0.68 | | 0.83 | -2.76 | | 0.51 | 0.42 | 0.71 |  |
|  | Leucine | 0.11 | 0.77 | -8.23 | | 0.16 | -15.13 | | **0.04** | 0.13 | 0.12 |  |
|  | Lysine | 0.04 | 0.93 | -15.96 | | **0.03** | -26.72 | | **0.005** | 0.06 | **0.02** |  |
|  | Methionine | 0.07 | 0.42 | -0.53 | | 0.69 | -1.92 | | 0.25 | 0.18 | 0.37 |  |
|  | Ornithine | -0.17 | 0.53 | -1.97 | | 0.63 | -0.56 | | 0.92 | 0.67 | 0.81 |  |
|  | Phenylalanine | 0.25 | 0.14 | -4.03 | | 0.12 | -10.48 | | **0.001** | **0.001** | **0.002** |  |
|  | Pipecolic acid | 0.02 | 0.55 | -0.05 | | 0.91 | -0.35 | | 0.54 | 0.39 | 0.69 |  |
|  | Proline | -0.33 | 0.76 | -6.57 | | 0.70 | -5.44 | | 0.81 | 0.93 | 0.93 |  |
|  | Serine | 0.12 | 0.70 | 5.88 | | 0.22 | 7.70 | | 0.21 | 0.63 | 0.42 |  |
|  | Taurine | 1.13 | **0.01** | 10.26 | | 0.16 | -1.25 | | 0.89 | **0.04** | **0.05** |  |
|  | Threonine | -0.50 | 0.18 | -0.46 | | 0.94 | 7.20 | | 0.34 | 0.10 | 0.26 |  |
|  | Trans-4-hydroxy-proline | -0.03 | 0.68 | -0.30 | | 0.78 | -0.04 | | 0.97 | 0.76 | 0.92 |  |
|  | Tyrosine | 0.06 | 0.78 | -1.06 | | 0.76 | -2.74 | | 0.54 | 0.55 | 0.80 |  |
|  | Valine | -0.32 | 0.63 | -11.22 | | 0.28 | -13.22 | | 0.32 | 0.81 | 0.54 |  |
| Acylcarnitines | Carnitine_C0 | -0.01 | 0.96 | -4.15 | | **0.02** | -6.67 | | **0.004** | 0.08 | **0.02** |  |
|  | Acetylcarnitine_C2 | 0.10 | **0.01** | 1.49 | | **0.03** | 0.79 | | 0.35 | 0.18 | **0.03** |  |
|  | Propionylcarnitine_C3 | -0.00001 | 0.99 | -0.03 | | 0.37 | -0.05 | | 0.26 | 0.48 | 0.52 |  |
|  | Butyrylcarnitine_C4 | 0.003 | 0.07 | 0.002 | | 0.94 | -0.05 | | 0.18 | **0.03** | 0.08 |  |
|  | Isovalerylcarnitine_C5 | 0.0004 | 0.39 | -0.01 | | 0.20 | -0.02 | | **0.02** | **0.03** | **0.05** |  |
|  | Hexanoylcarnitine_C6 | 0.001 | **0.03** | 0.01 | | **0.02** | 0.01 | | 0.20 | 0.33 | **0.04** |  |
|  | Octanoylcarnitine_C8 | 0.002 | 0.13 | 0.04 | | 0.05 | 0.04 | | 0.21 | 0.69 | 0.15 |  |
|  | Decanoylcarnitine_C10 | 0.004 | 0.09 | 0.09 | | **0.03** | 0.07 | | 0.16 | 0.65 | 0.09 |  |
|  | Lauroylcarnitine_C12 | 0.002 | **0.01** | 0.03 | | **0.01** | 0.02 | | 0.26 | 0.17 | **0.02** |  |
|  | Tetradecanoylcarnitine_C14 | 0.001 | **0.00004** | 0.01 | | **0.0004** | 0.01 | | 0.14 | **0.01** | **0.0001** |  |
|  | Palmitoylcarnitine_C16 | 0.002 | **0.00004** | 0.02 | | **0.01** | 0.001 | | 0.90 | **0.00** | **0.0002** |  |
|  | Stearoylcarnitine_C18 | 0.002 | **0.002** | 0.03 | | **0.002** | 0.02 | | 0.13 | 0.13 | **0.003** |  |
| TCA cycle | 2-oxo-glutarate | 0.04 | 0.29 | 0.80 | | 0.12 | 0.73 | | 0.27 | 0.88 | 0.31 |  |
|  | cis-Aconitate | 0.02 | **0.0001** | 0.27 | | **0.002** | 0.10 | | 0.33 | **0.01** | **0.0002** |  |
|  | Citrate | 1.50 | **0.002** | 23.15 | | **0.002** | 13.96 | | 0.14 | 0.12 | **0.003** |  |
|  | Glucose | 31.56 | 0.19 | 231.39 | | 0.55 | -124.04 | | 0.80 | 0.24 | 0.42 |  |
|  | Isocitrate | 0.06 | **0.001** | 0.49 | | 0.10 | -0.14 | | 0.70 | **0.01** | **0.01** |  |
|  | Lactate | 3.45 | 0.75 | 103.54 | | 0.53 | 114.12 | | 0.59 | 0.94 | 0.82 |  |
|  | Pyruvate | 0.17 | 0.83 | -2.97 | | 0.82 | -7.61 | | 0.64 | 0.65 | 0.88 |  |
| Tryptophan pathway | 3-hydroxy-kynurenine | 0.0002 | 0.37 | -0.002 | | 0.69 | -0.01 | | 0.22 | 0.14 | 0.31 |  |
|  | Kynurenic acid | 0.00003 | 0.92 | -0.01 | | 0.10 | -0.01 | | **0.03** | 0.14 | 0.08 |  |
|  | Kynurenine | 0.01 | 0.21 | -0.05 | | 0.71 | -0.24 | | 0.14 | 0.06 | 0.16 |  |
|  | Oxoadipic acid | -0.002 | 0.38 | 0.01 | | 0.64 | 0.05 | | 0.20 | 0.14 | 0.30 |  |
|  | Quinolinate | 0.01 | **0.04** | 0.05 | | 0.47 | -0.06 | | 0.48 | **0.04** | 0.10 |  |
|  | Tryptophan | -0.33 | 0.08 | -7.58 | | **0.01** | -7.14 | | 0.06 | 0.85 | **0.04** |  |

Conc, concentration; β, beta-coefficient; P_β_, P-value of beta coefficient; P_model_, P-value of model. ‘Metabolite concentration = age’ represents the association/correlation between the metabolite concentrations and age. ‘Metabolite concentration = group’ represents the AD vs control group differences similar to the presented t-tests. ‘Metabolite concentration = group + age’ represents the group differences adjusted for the confounding effect of age.

1. **CSF data**

|  | Metabolites | Metabolite = age | | | Metabolite = group | | Metabolite = group + age | | | | |
| --- | --- | --- | --- | --- | --- | --- | --- | --- | --- | --- | --- |
|  |  | **β_age_** | **P_β_** | **β_group_** | | **P_β_** | **β** | **P_β_** | **P_age_** | **P_model_** |  |
| Amino acids | Arginine | -0.07 | 0.18 | 0.15 | | 0.87 | 1.31 | 0.22 | 0.07 | 0.19 |  |
|  | Asparagine | -0.02 | 0.55 | -0.57 | | 0.37 | -0.53 | 0.50 | 0.94 | 0.67 |  |
|  | Citrulline | 0.003 | 0.82 | 0.25 | | 0.23 | 0.35 | 0.19 | 0.54 | 0.40 |  |
|  | Creatinine | 0.64 | **0.0004** | 11.76 | | **0.00002** | 9.04 | **0.01** | 0.16 | **0.00004** |  |
|  | Glutamine | 1.14 | 0.27 | 16.96 | | 0.29 | 9.97 | 0.62 | 0.56 | 0.49 |  |
|  | Histidine | -0.09 | **0.01** | -1.44 | | **0.01** | -0.90 | 0.19 | 0.18 | **0.02** |  |
|  | Isoleucine | 0.01 | 0.68 | -0.03 | | 0.94 | -0.19 | 0.69 | 0.56 | 0.84 |  |
|  | Leucine | -0.02 | 0.78 | -0.92 | | 0.30 | -1.20 | 0.28 | 0.67 | 0.53 |  |
|  | Lysine | -0.11 | 0.17 | -2.56 | | **0.04** | -2.38 | 0.12 | 0.84 | 0.12 |  |
|  | Ornithine | -0.05 | 0.15 | -0.08 | | 0.88 | 0.54 | 0.38 | 0.09 | 0.24 |  |
|  | Phenylalanine | 0.02 | 0.50 | -0.10 | | 0.83 | -0.46 | 0.44 | 0.32 | 0.59 |  |
|  | Pipecolic acid | 0.001 | 0.19 | 0.01 | | 0.32 | 0.004 | 0.79 | 0.37 | 0.41 |  |
|  | Proline | -0.01 | 0.43 | -0.12 | | 0.27 | -0.11 | 0.44 | 0.86 | 0.54 |  |
|  | Serine | -0.02 | 0.84 | -0.05 | | 0.97 | 0.15 | 0.92 | 0.83 | 0.97 |  |
|  | Taurine | 0.02 | 0.65 | 0.25 | | 0.64 | 0.17 | 0.80 | 0.83 | 0.88 |  |
|  | Threonine | -0.12 | 0.21 | 1.37 | | 0.37 | 3.91 | **0.04** | **0.02** | **0.05** |  |
|  | trans-4-hydroxy-Proline | -0.001 | 0.88 | -0.02 | | 0.73 | -0.03 | 0.75 | 0.95 | 0.94 |  |
|  | Tyrosine | 0.01 | 0.71 | -0.19 | | 0.70 | -0.47 | 0.45 | 0.46 | 0.71 |  |
|  | Valine | -0.01 | 0.86 | -0.62 | | 0.60 | -0.77 | 0.60 | 0.86 | 0.86 |  |
| Acylcarnitines | Carnitine_C0 | -0.005 | 0.41 | 0.01 | | 0.91 | 0.09 | 0.45 | 0.27 | 0.54 |  |
|  | Acetylcarnitine_C2 | 0.001 | 0.66 | 0.05 | | 0.36 | 0.05 | 0.42 | 0.89 | 0.65 |  |
|  | Propionylcarnitine_C3 | -0.0001 | 0.74 | 0.0001 | | 0.98 | 0.001 | 0.79 | 0.68 | 0.92 |  |
|  | Butyrylcarnitine_C4 | 0.0003 | 0.14 | 0.003 | | 0.43 | -0.001 | 0.91 | 0.21 | 0.33 |  |
|  | Isovalerylcarnitine_C5 | 0.000003 | 0.97 | -0.0005 | | 0.69 | -0.001 | 0.60 | 0.73 | 0.87 |  |
|  | Hexanoylcarnitine_C6 | 0.00001 | 0.49 | 0.0002 | | 0.11 | 0.0003 | 0.14 | 0.75 | 0.26 |  |
|  | Octanoylcarnitine_C8 | 0.000002 | 0.85 | 0.0003 | | 0.06 | 0.0004 | **0.03** | 0.23 | 0.08 |  |
|  | Decanoylcarnitine_C10 | 0.000003 | 0.52 | 0.0002 | | **0.04** | 0.0002 | **0.04** | 0.48 | 0.10 |  |
|  | Lauroylcarnitine_C12 | 0.0000002 | 0.92 | 0.00002 | | 0.52 | 0.00002 | 0.47 | 0.72 | 0.76 |  |
| TCA cycle | 2-oxo-glutarate | 0.005 | 0.06 | 0.09 | | **0.02** | 0.07 | 0.14 | 0.53 | 0.06 |  |
|  | cis-Aconitate | 0.002 | 0.65 | 0.12 | | 0.09 | 0.15 | 0.08 | 0.49 | 0.19 |  |
|  | Citrate | 2.06 | 0.11 | 41.37 | | **0.04** | 34.68 | 0.17 | 0.65 | 0.11 |  |
|  | Fructose | 0.75 | 0.41 | 16.96 | | 0.23 | 15.64 | 0.38 | 0.90 | 0.48 |  |
|  | Glucose | 1.52 | 0.88 | -72.09 | | 0.64 | -134.32 | 0.48 | 0.59 | 0.77 |  |
|  | Isocitrate | -0.001 | 0.92 | 0.12 | | 0.45 | 0.21 | 0.31 | 0.49 | 0.59 |  |
|  | Lactate | 5.97 | 0.10 | 49.03 | | 0.39 | -10.00 | 0.89 | 0.17 | 0.26 |  |
|  | Malate | 0.003 | 0.33 | 0.10 | | 0.08 | 0.10 | 0.14 | 0.92 | 0.21 |  |
|  | Pyruvate | 0.19 | 0.63 | 5.59 | | 0.37 | 5.91 | 0.45 | 0.95 | 0.66 |  |
| Tryptophan pathway | Anthranillic acid | -0.001 | 0.15 | -0.001 | | 0.92 | 0.01 | 0.33 | 0.08 | 0.22 |  |
|  | Kynurenic acid | 0.0001 | **0.001** | 0.001 | | 0.05 | -0.00003 | 0.96 | **0.01** | **0.003** |  |
|  | Quinolinate | 0.001 | **0.02** | 0.01 | | **0.04** | 0.004 | 0.41 | 0.17 | **0.05** |  |
|  | Tryptophan | -0.004 | 0.56 | -0.03 | | 0.78 | 0.01 | 0.93 | 0.60 | 0.84 |  |

Conc, concentration; β, beta-coefficient; P_β_, P-value of beta coefficient; P_model_, P-value of model. ‘Metabolite concentration = age’ represents the association/correlation between the metabolite concentrations and age. ‘Metabolite concentration = group’ represents the AD vs control group differences similar to the presented t-tests. ‘Metabolite concentration = group + age’ represents the group differences adjusted for the confounding effect of age.

**Table S9.** Results of the ANOVA analysis for the interaction between the AD effect (group) and ApoE4 effect, Table A) in plasma and Table B) in CSF

1. **Plasma**

|  | metabolite | P_group_ | P_ApoE4_ | P_Interactopm_ |
| --- | --- | --- | --- | --- |
| Amino acids | Arginine | 0.52 | 0.50 | 0.36 |
|  | Asparagine | 0.63 | **0.01** | 0.88 |
|  | Citrulline | 0.95 | 0.73 | 0.76 |
|  | Creatinine | 0.85 | **0.004** | **0.02** |
|  | Glutamic acid | 0.86 | 0.30 | 0.18 |
|  | Glutamine | 0.07 | 0.27 | 0.32 |
|  | Glycine | 0.88 | 0.38 | 0.96 |
|  | Histidine | **0.02** | 0.63 | 0.69 |
|  | Isoleucine | 0.83 | **0.04** | 0.99 |
|  | Leucine | 0.15 | 0.06 | 0.56 |
|  | Lysine | **0.03** | 0.42 | 0.39 |
|  | Methionine | 0.69 | 0.16 | 0.96 |
|  | Ornithine | 0.64 | 0.33 | 0.90 |
|  | Oxoadipic acid | 0.65 | 0.61 | 0.35 |
|  | Phenylalanine | 0.13 | 0.52 | 0.57 |
|  | Pipecolic acid | 0.91 | 0.67 | 0.13 |
|  | Proline | 0.70 | **0.05** | 0.71 |
|  | Serine | 0.22 | 0.70 | 0.94 |
|  | Taurine | 0.16 | 0.61 | 0.14 |
|  | Threonine | 0.94 | 0.69 | 0.23 |
|  | Trans-4-hydroxy-proline | 0.78 | 0.81 | 0.99 |
|  | Tyrosine | 0.76 | 0.41 | 0.27 |
|  | Valine | 0.27 | 0.10 | 0.88 |
| Acylcarnitines | Carnitine_C0 | **0.02** | 0.20 | 0.45 |
|  | Acetylcarnitine_C2 | **0.03** | 0.83 | 0.89 |
|  | Propionylcarnitine_C3 | 0.37 | 0.12 | 0.60 |
|  | Butyrylcarnitine_C4 | 0.94 | 0.94 | 0.20 |
|  | Isovalerylcarnitine_C5 | 0.20 | 0.13 | 0.50 |
|  | Hexanoylcarnitine_C6 | **0.02** | 0.48 | 0.63 |
|  | Octanoylcarnitine_C8 | 0.06 | 0.39 | 0.84 |
|  | Decanoylcarnitine_C10 | **0.04** | 0.74 | 0.91 |
|  | Lauroylcarnitine_C12 | **0.01** | 0.44 | 0.22 |
|  | Tetradecanoylcarnitine_C14 | **0.0003** | 0.50 | 0.17 |
|  | Palmitoylcarnitine_C16 | **0.01** | 0.66 | 0.11 |
|  | Stearoylcarnitine_C18 | **0.002** | 0.81 | 0.12 |
| TCA cycle | 2-oxo-glutarate | 0.13 | 0.46 | 0.82 |
|  | cis-Aconitate | **0.002** | 0.52 | 0.29 |
|  | Citrate | **0.003** | 0.90 | 0.33 |
|  | Glucose | 0.55 | 0.27 | 0.78 |
|  | Isocitrate | 0.10 | 0.57 | 0.69 |
|  | Lactate | 0.53 | 0.13 | 0.67 |
|  | Pyruvate | 0.81 | 0.08 | 0.28 |
| Tryptophan pathway | 3-hydroxy-kynurenine | 0.69 | 0.59 | 0.39 |
|  | Kynurenic acid | 0.09 | 0.16 | 0.58 |
|  | Kynurenine | 0.71 | 0.50 | 0.88 |
|  | Tryptophan | **0.01** | 0.46 | 0.18 |
|  | Quinolinate | 0.48 | 0.65 | 0.61 |

P values derived from ANOVA model ‘metabolite concentration = group * ApoE4 status’, in which both ‘group’ and ‘ApoE4’ are binary variables.

1. **CSF**

|  | metabolite | P_group_ | P_ApoE4_ | P_Interactopm_ |
| --- | --- | --- | --- | --- |
| Amino acids | Arginine | 0.87 | 0.58 | 0.98 |
|  | Asparagine | 0.33 | **0.01** | **0.005** |
|  | Citrulline | 0.23 | 0.77 | 0.38 |
|  | Creatinine | **0.00002** | 0.66 | 0.76 |
|  | Glutamine | 0.30 | 0.51 | 0.65 |
|  | Histidine | **0.01** | 0.88 | 0.87 |
|  | Isoleucine | 0.94 | 0.14 | 0.90 |
|  | Leucine | 0.30 | 0.12 | 0.71 |
|  | Lysine | **0.04** | 0.48 | 0.80 |
|  | Ornithine | 0.88 | 0.39 | 0.49 |
|  | Phenylalanine | 0.83 | 0.63 | 0.73 |
|  | Pipecolic acid | 0.32 | 0.83 | 0.23 |
|  | Proline | 0.27 | 0.24 | 0.31 |
|  | Serine | 0.97 | 0.15 | 0.46 |
|  | Taurine | 0.63 | 0.06 | 0.64 |
|  | Threonine | 0.37 | 0.16 | 0.85 |
|  | trans-4-hydroxy-Proline | 0.73 | 0.42 | 0.39 |
|  | Tyrosine | 0.71 | 0.98 | 0.43 |
|  | Valine | 0.60 | 0.15 | 0.93 |
| Acylcarnitines | Carnitine_C0 | 0.91 | 0.40 | 0.25 |
|  | Acetylcarnitine_C2 | 0.36 | 0.61 | 0.73 |
|  | Propionylcarnitine_C3 | 0.98 | 0.21 | 0.47 |
|  | Butyrylcarnitine_C4 | 0.43 | 0.77 | 0.83 |
|  | Isovalerylcarnitine_C5 | 0.69 | 0.18 | 0.99 |
|  | Hexanoylcarnitine_C6 | 0.11 | 0.49 | 0.94 |
|  | Octanoylcarnitine_C8 | 0.06 | 0.44 | 0.74 |
|  | Decanoylcarnitine_C10 | **0.04** | 0.71 | 0.34 |
|  | Lauroylcarnitine_C12 | 0.52 | 0.58 | 0.86 |
| TCA cycle | 2-oxo-glutarate | **0.02** | 0.12 | 0.62 |
|  | cis-Aconitate | 0.10 | 0.70 | 0.78 |
|  | Citrate | **0.04** | 0.87 | 0.82 |
|  | Fructose | 0.23 | 0.19 | 0.39 |
|  | Glucose | 0.64 | 0.43 | 0.95 |
|  | Isocitrate | 0.45 | 0.44 | 0.76 |
|  | Lactate | 0.40 | 0.53 | 0.64 |
|  | Malate | 0.08 | 0.89 | 0.70 |
|  | Pyruvate | 0.37 | 0.64 | 0.85 |
| Tryptophan pathway | Anthranillic acid | 0.93 | 0.59 | 1.00 |
|  | Kynurenic acid | **0.05** | 0.07 | 0.12 |
|  | Tryptophan | 0.78 | 0.59 | 0.51 |
|  | Quinolinate | **0.04** | 1.00 | 0.69 |

P values derived from ANOVA model ‘metabolite concentration = group * ApoE4 status’, in which both ‘group’ and ‘ApoE4’ are binary variables.


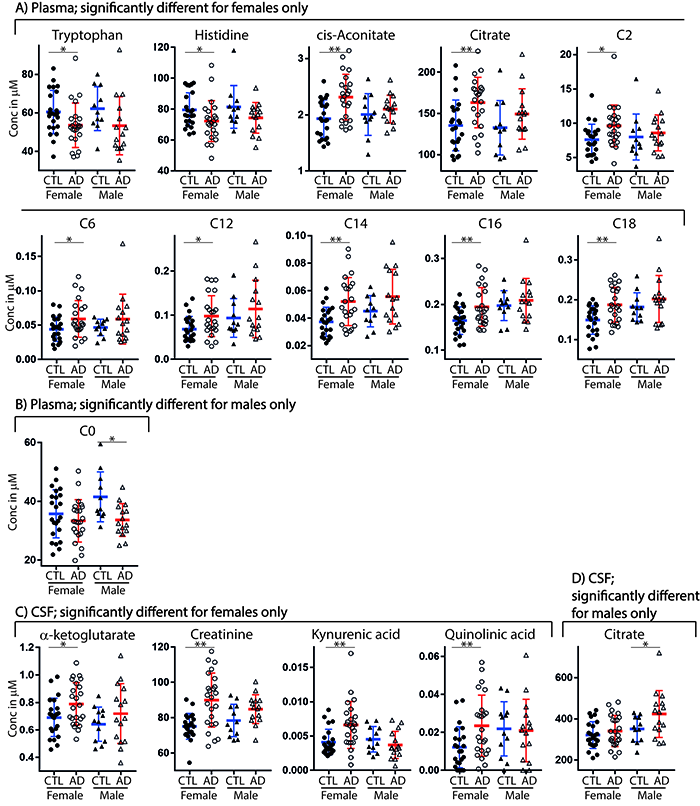


**Figure S1.** Sex-related significant differences at the metabolite level, measured in plasma a) and b), and measured in CSF c) and d).
