# Supplementary material for: Systemic and central nervous system metabolic alterations in Alzheimer’s disease
Source: Alzheimers Res Ther. 2019 Nov 28;11:93. doi: 10.1186/s13195-019-0551-7 (PMC6883620; doi:10.1186/s13195-019-0551-7)
Supplement: Supplementary file 2 — Additional file 2. Supplemental Methods. [file 13195_2019_551_MOESM2_ESM.docx]

**Supplemental Methods**

**1.1 AD diagnosis and cognitive assessments**

The diagnosis of MCI or mild dementia of AD type was based on neuropsychological and clinical evaluation made by a consensus conference of neuropsychologists, psychiatrists, and/or neurologists prior to inclusion into the study, as described elsewhere [1]. MCI was diagnosed according to consensus recommendations [2]; i.e. the participants in this group had memory and/or impairment in another cognitive domain (see below), and a Clinical Dementia Rating (CDR) [3] score of >0.5. The diagnosis of mild dementia was based on the clinical diagnostic criteria for probable dementia due to AD according to recommendations from the National Institute on Aging and Alzheimer’s Association [4] and the DSM-IV criteria for dementia of the Alzheimer type [5]. The participants without cognitive impairment had no history or evidence of cognitive deficits, and their CDR score was 0. Comprehensive neuropsychological tests were used to assess cognitive performance in the domains of memory, language/verbal fluency, executive functions, and visual-constructive functions [1]. The Mini Mental State Examination [6] was used to assess participants’ global cognitive performance.. All tests and scales are validated and widely used in the field.

**1.2 Sample collection**

CSF and plasma samples were obtained as previously described [1], details are described in the supplemental material. In brief, 10–12mL fasted CSF samples were collected using lumbar puncture, using a standardized technique with a 22-gauge “atraumatic” spinal needle [7]. Afterwards, fasting EDTA blood samples were collected and plasma was obtained by centrifugation (6°C, 1100g, 12min). Both biofluids were stored at −80 °C before further use.

**1.3 CSF AD biomarkers, APOE and biochemical markers**

CSF AD biomarkers Aβ1–42, tau and pTau-181 were measured using ELISA (Fujirebio, Ghent, Belgium) and the APOE genotype (i.e. ɛ4 allele status) was determined, as previously described [8]. A pathological CSF AD biomarker profile was defined as a pTau-181/Aβ1-42 ratio >0.078 as previously described [9] and in line with published recommendations [10, 11]. Other biochemical markers (glucose, blood lipids, CSF/serum albumin ratio) were obtained from plasma and CSF using routine methods at the CHUV Clinical Chemistry Laboratory.

**1.4 Next generation metabolic profiling - Untargeted & Targeted Metabolite Analysis.**

***1.4.1 Materials.*** Relevant standards were purchased from Sigma and stable isotope labelled internal standards (D-, 13C- or 15N-labelled) were purchased from Cambridge Isotope Labs (Tweksbury, US) and are listed in Supplementary table 7. All other chemicals (i.e. solvents and reagents) were of LC-MS grade and purchased from Sigma Aldrich.

***1.4.2 Untargeted profiling.*** Plasma and CSF samples (100μL) were extracted by the addition of 400μL of ice-cold MeOH:ACN (1:1, v/v) to achieve a MeOH:ACN:H_2_O ratio of 2:2:1 (v/v). Samples were vortexed for 30s, incubated for 1h at −20°C, and centrifuged at 13,000rpm for 15min at 4°C. The resulting supernatant was evaporated to dryness using a SpeedVac (LabConco, Missouri, US). The dry extracts were then reconstituted in 100μL of H_2_O:MeOH:ACN (2:1:1, v/v), sonicated for 1min and centrifuged 10min at 13,000rpm at 4°C to remove the insoluble debris. The diluted supernatants (40x for plasma and 10x for CSF) were transferred to HPLC vials for LC-MS analysis. Samples were then randomized and analyzed

by ultra-high performance liquid chromatography - electrospray ionization high-resolution mass spectrometry (UHPLC-ESI-HRMS) using the 6550 iFunnel Q-TOF MS interfaced with 1290 UHPLC (Agilent Technologies, Basel, CH) as previously described [12]. The data were processed using XCMS Online [13, 14] and the data table containing peak abundances of all detected metabolite features was further processed in R for signal drift correction using a locally quadratic (loess) regression model [15, 16]. Features showing high analytical variability (CV > 30%) were considered as noise and thus removed from the dataset. The Mann-Whitney test was used to select the metabolite features whose levels were altered significantly between two groups (control vs. AD). Putative identification (using accurate mass) of these features was done automatically in XCMS Online through the integrated METLIN metabolite database search. Metabolite identities were further validated with tandem MS experiments as previously described [12].

***1.4.3 Broad scale targeted profiling.*** In parallel with untargeted profiling, broad scale targeted screening with a focus on intermediates involved in multiple central carbon pathways (242 metabolites) was performed on plasma and CSF extracts using a 6495 iFunnel triple quadrupole system (QqQ, Agilent Technologies, Basel, CH) to gain sensitivity (as compared to untargeted profiling). The data was acquired in dynamic multiple reaction monitoring mode (dMRM, cycle time 600ms). The collision energies and MS/MS transitions were optimized for each metabolite of interest using the Mass Spectrometry Metabolite Library of Standards (MSMLS, Sigma, Buchs, CH, Supplemental table 7). ESI source conditions were set as follows: dry gas temperature 290°C, nebulizer 35psi and flow 14L/min, sheath gas temperature 350°C and flow 12L/min, nozzle voltage 0V, and capillary voltage +2000V in positive and -2000V in negative mode. Negative electrospray ionization (ESI - MS) mode was performed using a SeQuant ZIC-pHILIC column (100x2.1mm I.D., 5μm) with a SeQuant ZIC-pHILIC guard column (20x2.1mm I.D., 5μm (Merck, Darmstadt, Germany) at an operation temperature of 30°C. Mobile phase A was composed of 20mM ammonium acetate and 20mM ammonium hydroxide in H_2_O (pH 9.3) and mobile phase B was 100% acetonitrile, and the sample injection volume was 2µl. The linear gradient elution started at 90% B (0-1.5min), decreased to 50% B (8-11min), further decreased to 45% B (12-15min) and restored to 90% (15-16min) with a re-equilibration step for 9 minutes at a flow rate of 300μL/min.

In positive electrospray ionization (ESI + MS) mode, using a Acquity BEH Amide, 1.7 μm, 100 mm × 2.1 mm I.D. column (Waters, Massachusetts, US), the mobile phase was composed of A = 20 mM ammonium formate and 0.1 % FA in water and B = 0.1 % FA in ACN. A linear gradient elution from 95% B (0-1.5 min) to 45% B (17 -19 min) was applied followed by 5min for column re-equilibration in the initial gradient conditions. The flow rate was 400 μL/min, column temperature 25 °C and sample injection volume 2µl.

Data processing was done using MassHunter Quantitative Analysis (for QqQ, version B.07.01/ Build 7.1.524.0, Agilent Technologies) and the data table containing peak areas of all measured metabolites was further processed in R for signal drift correction applying a locally quadratic (loess) regression model [15, 16] on QC samples (i.e. pooled samples analyzed periodically across the run). Metabolites with CV > 20% were discarded. Non-parametric, Mann-Whitney U-test was used to assign the statistically significant differences between AD patients and control subjects (R, version 3.4.1, http://www.R-project.org/).

***1.4.4 Targeted quantification of TCA cycle intermediates, tryptophan breakdown products and other amino acids and acylcarnitines.*** Targeted quantification was performed using the 6495 QqQ mass spectrometer interfaced with the 1290 UHPLC system, operated in the dMRM mode. Samples were prepared by mixing an aliquot of calibrator (5μL), plasma (5μL) or CSF (10μL) with 250μL of the ice-cold internal standard solution (in 100% acetonitrile) and H_2_O was added to reach a final volume of 300μL. Samples were centrifuged and supernatant was directly injected for LC-MS/MS analysis (Supplemental Table 4). Quantification was performed in both positive and negative ionization mode, using acidic or basic LC conditions depending on the metabolite ionization efficiency. Where available, stable isotope labeled internal standards were used to determine the response factor, otherwise concentrations were reported with an external calibration curve, following the signal drift correction. Data processing was done using MassHunter Quantitative Analysis. Peak area integration was manually curated and concentrations were reported by selecting a 6-point portion of the linear standard curves relevant for the observed concentrations. Group comparisons were done using the unpaired T-test.

***1.4.5. Pathway analyses.*** Pathway analysis were performed using MetaboAnalyst 3.0 [17] and the human pathways from the Homo sapiens Kyoto Encyclopedia of Genes and Genomes (KEGG) [18] database and the Homo sapiens Small Molecule Pathway Database (SMPDB) [19] were used as the source of pathway topologies to deduce pathways of interest for the absolute quantification method. The data was log-transformed and normalized using auto-scaling. The level of significance for Metabolite Set Enrichment Analysis (MSEA) was calculated using the probability test (*globaltest* algorithm)[20] that the set of functionally-related metabolites (implicated in specific pathways) is represented within a defined list of measured metabolites more than expected by a random chance. Pathway impact has been calculated as the sum of the importance measures (i.e. centrality measure within a given metabolic network) of the matched metabolites normalized by the sum of the importance measures of all metabolites in each pathway[21].

**1.5 Statistical analysis**

Gender differences (female vs. male) were assessed separately in AD patients and controls using unpaired t-tests, for those metabolites that were significantly different in the group comparisons. To further explore whether the observed alterations in CSF originate systemically or are produced in the CNS, the ratio of concentration between CSF and plasma was calculated and evaluated for all metabolites between AD patients and controls (t-test). To investigate the impact of BBB permeability on the observed alterations the metabolite concentrations in CSF were correlated with the Qalb (ratio of CSF albumin concentration and plasma albumin concentration) separately in AD patients and control subjects, using Pearson correlations. Lastly, the influence of age on the group differences was evaluated using a linear regression model (Metabolite concentration = group (AD and control) + age), from which the beta-coefficient of group, the p-values and confidence intervals were evaluated. To evaluate the contribution of APOE4 an ANOVA with an interaction term was performed (Metabolite concentration = group (AD and control) * APOE4 (yes or no)) and the p-values for the interaction were evaluated.

**References**

[1] Popp J, Oikonomidi A, Tautvydaite D, Dayon L, Bacher M, Migliavacca E, et al. Markers of neuroinflammation associated with Alzheimer's disease pathology in older adults. Brain Behav Immun. 2017;62:203-11.

[2] Winblad B, Palmer K, Kivipelto M, Jelic V, Fratiglioni L, Wahlund LO, et al. Mild cognitive impairment--beyond controversies, towards a consensus: report of the International Working Group on Mild Cognitive Impairment. J Intern Med. 2004;256:240-6.

[3] Morris JC. The Clinical Dementia Rating (CDR): current version and scoring rules. Neurology. 1993;43:2412-4.

[4] McKhann GM, Knopman DS, Chertkow H, Hyman BT, Jack CR, Jr., Kawas CH, et al. The diagnosis of dementia due to Alzheimer's disease: recommendations from the National Institute on Aging-Alzheimer's Association workgroups on diagnostic guidelines for Alzheimer's disease. Alzheimers Dement. 2011;7:263-9.

[5] Association AP. Diagnostic and Statistical Manual of Mental Disorders. 2000;Fourth edition.

[6] Folstein MF, Folstein SE, McHugh PR. "Mini-mental state". A practical method for grading the cognitive state of patients for the clinician. J Psychiatr Res. 1975;12:189-98.

[7] Popp J, Riad M, Freymann K, Jessen F. [Diagnostic lumbar puncture performed in the outpatient setting of a memory clinic. Frequency and risk factors of post-lumbar puncture headache]. Nervenarzt. 2007;78:547-51.

[8] Tautvydaite D, Antonietti JP, Henry H, von Gunten A, Popp J. Relations between personality changes and cerebrospinal fluid biomarkers of Alzheimer's disease pathology. J Psychiatr Res. 2017;90:12-20.

[9] Dayon L, Guiraud SP, Corthesy J, Da Silva L, Migliavacca E, Tautvydaite D, et al. One-carbon metabolism, cognitive impairment and CSF measures of Alzheimer pathology: homocysteine and beyond. Alzheimers Res Ther. 2017;9:43.

[10] Duits FH, Teunissen CE, Bouwman FH, Visser PJ, Mattsson N, Zetterberg H, et al. The cerebrospinal fluid "Alzheimer profile": easily said, but what does it mean? Alzheimers Dement. 2014;10:713-23 e2.

[11] Molinuevo JL, Blennow K, Dubois B, Engelborghs S, Lewczuk P, Perret-Liaudet A, et al. The clinical use of cerebrospinal fluid biomarker testing for Alzheimer's disease diagnosis: a consensus paper from the Alzheimer's Biomarkers Standardization Initiative. Alzheimers Dement. 2014;10:808-17.

[12] Gallart-Ayala H, Konz I, Mehl F, Teav T, Oikonomidi A, Peyratout G, et al. A global HILIC-MS approach to measure polar human cerebrospinal fluid metabolome: Exploring gender-associated variation in a cohort of elderly cognitively healthy subjects. Anal Chim Acta. 2018.

[13] Gowda H, Ivanisevic J, Johnson CH, Kurczy ME, Benton HP, Rinehart D, et al. Interactive XCMS Online: simplifying advanced metabolomic data processing and subsequent statistical analyses. Anal Chem. 2014;86:6931-9.

[14] Tautenhahn R, Cho K, Uritboonthai W, Zhu Z, Patti GJ, Siuzdak G. An accelerated workflow for untargeted metabolomics using the METLIN database. Nat Biotechnol. 2012;30:826-8.

[15] van der Kloet FM, Bobeldijk I, Verheij ER, Jellema RH. Analytical error reduction using single point calibration for accurate and precise metabolomic phenotyping. J Proteome Res. 2009;8:5132-41.

[16] Dunn WB, Broadhurst D, Begley P, Zelena E, Francis-McIntyre S, Anderson N, et al. Procedures for large-scale metabolic profiling of serum and plasma using gas chromatography and liquid chromatography coupled to mass spectrometry. Nat Protoc. 2011;6:1060-83.

[17] Xia J, Wishart DS. Using MetaboAnalyst 3.0 for Comprehensive Metabolomics Data Analysis. Curr Protoc Bioinformatics. 2016;55:14 0 1- 0 91.

[18] Kanehisa M, Goto S. KEGG: Kyoto Encyclopedia of Genes and Genomes. Nucleic Acids Research. 2000;28:27-30.

[19] Frolkis A, Knox C, Lim E, Jewison T, Law V, Hau DD, et al. SMPDB: The Small Molecule Pathway Database. Nucleic Acids Res. 2010;38:D480-7.

[20] Xia J, Wishart DS. MSEA: a web-based tool to identify biologically meaningful patterns in quantitative metabolomic data. Nucleic Acids Res. 2010;38:W71-W7.

[21] Wishart DS, Xia J. MetPA: a web-based metabolomics tool for pathway analysis and visualization. Bioinformatics. 2010;26:2342-4.
